# Biochemical and renal-metabolic profiles distinguishing primary aldosteronism from essential hypertension: a systematic review and meta-analysis with implications for endocrine hypertension screening

**DOI:** 10.3389/fendo.2026.1916237

**Published:** 2026-09-15

**Authors:** Xuan Zhao, Caiyan Zhang, Chunxiu Yi, Xinyi Liu, Shujun Ding, Wen Zhang, Yajing Zhao

**Affiliations:** Fuwai Yunnan Hospital, Chinese Academy of Medical Sciences, Affiliated Cardiovascular Hospital of Kunming Medical University, Kunming, Yunnan, China

**Keywords:** primary aldosteronism, essential hypertension, serum potassium, serum sodium, albuminuria, meta-analysis

## Abstract

**Introduction:**

To characterize electrolyte and renal-metabolic profiles in primary aldosteronism (PA) compared with essential hypertension (EH), and to examine differences between unilateral/lateralizing and bilateral PA.

**Methods:**

PubMed, Embase, the Cochrane Library, and Web of Science were searched from inception through 13 July 2026. Random-effects meta-analyses pooled standardized mean differences (SMD; Hedges' g), supplemented by clinical-unit mean-difference analyses, subgroup analyses, sensitivity analyses, and study-level meta-regression.

**Results:**

A total of 74 observational studies involving 26,143 participants were included. Compared with hypertensive controls, PA was associated with lower serum potassium (SMD = −1.15, 95% CI −1.27 to −1.02), higher serum sodium (SMD = 0.51, 95% CI 0.38 to 0.63), and lower uric acid (SMD = −0.23, 95% CI −0.35 to −0.12), whereas creatinine, estimated glomerular filtration rate (eGFR), and blood urea nitrogen (BUN) did not differ significantly. Corresponding PA-minus-control mean differences were −0.49 mmol/L for potassium, +1.20 mmol/L for sodium, and −20.7 μmol/L for uric acid. Potassium was lower in unilateral/lateralizing than in bilateral PA (SMD = −0.85). The potassium difference versus controls was greater in studies with high plasma aldosterone concentration (PAC) than in those with low PAC (SMD −1.44 vs. −1.03; p for subgroup difference = 0.010), although continuous PAC meta-regression was not significant. UACR/ACR and urinary albumin excretion were higher in PA (SMD = 0.49 and 0.73).

**Discussion:**

PA shows a persistent electrolyte phenotype and higher albuminuria despite similar cross-sectional filtration markers. These findings support renal assessment with UACR alongside routine filtration measures and reinforce that PA screening should not depend on overt hypokalemia alone.

**Systematic Review Registration:**

https://www.crd.york.ac.uk/PROSPERO/, identifier CRD420261286832.

## 1 Introduction

Primary aldosteronism (PA) comprises disorders characterized by autonomous aldosterone secretion and is the most common cause of secondary hypertension (1, 2). PA affects approximately 5%–10% of the general hypertensive population and 15%–20% of patients with resistant hypertension (3–5). Even among patients with stage 1 hypertension, biochemically confirmed PA may be present in approximately 16%, underscoring substantial under-recognition (4). Current clinical terminology distinguishes unilateral/lateralizing PA from bilateral PA because this distinction guides treatment: unilateral/lateralizing disease may be amenable to adrenalectomy, whereas bilateral disease is generally managed medically (1, 2, 6–8). Historical labels such as aldosterone-producing adenoma (APA) and idiopathic hyperaldosteronism (IHA) remain common in the literature but do not fully encompass the contemporary clinical classification.

Aldosterone increases distal sodium reabsorption and potassium secretion through mineralocorticoid receptor signaling (9, 10). The wider use of aldosterone-to-renin ratio (ARR) screening, together with recognition of a continuum of renin-independent aldosterone production, has expanded the PA phenotype beyond the classic hypokalemic presentation (1, 2, 4, 6, 8, 11–14). This changing case spectrum complicates interpretation of older estimates of biochemical severity and target-organ involvement. Previous meta-analyses have examined cardiovascular, skeletal, glycemic, and renal outcomes (15–19), but important uncertainties remain. In particular, a 2020 meta-analysis of 22 studies reported higher estimated glomerular filtration rate (eGFR) in PA than in essential hypertension (EH) (18), whereas subsequent large studies have broadened the evidence base. Urinary albumin and serum uric acid may provide complementary information on renal hemodynamics and tubular handling, yet these outcomes have not been integrated with electrolyte and filtration-marker data (20–22).

We therefore synthesized electrolyte, renal-metabolic, and urinary albumin outcomes in PA versus EH, examined differences between unilateral/lateralizing and bilateral PA, and assessed whether study characteristics and hormonal profiles helped explain variation across studies.

Because this review evaluated a disease exposure rather than a therapeutic intervention, the question was structured using an exposure-based PICOS/PECOS framework: adults with hypertension constituted the population; biochemically confirmed PA was the exposure; EH, or bilateral PA in subtype analyses, was the comparator; electrolyte, renal-metabolic, urinary albumin, bicarbonate, and hormonal measures were the outcomes; and eligible designs were observational cross-sectional, case-control, and cohort studies.

## 2 Materials and methods

### 2.1 Search strategy

A systematic search of PubMed, Embase, the Cochrane Library, and Web of Science was conducted from database inception through July 13, 2026. Complete search strings for all databases appear in **Supplementary Table S1**. Search terms combined PA-related terms (“primary aldosteronism”, “Conn syndrome”, “aldosterone-producing adenoma”, and “idiopathic hyperaldosteronism”) with contemporary subtype terms (“unilateral”, “lateralizing”, “lateralized”, “lateralised”, and “bilateral”) and outcome-specific terms for potassium, sodium, creatinine, glomerular filtration rate, blood urea nitrogen, uric acid, albuminuria/proteinuria, bicarbonate, and alkalosis. No language restrictions were applied. Reference lists of all included studies and relevant systematic reviews were also hand-searched.

This systematic review and meta-analysis was conducted and reported in accordance with the Preferred Reporting Items for Systematic Reviews and Meta-Analyses (PRISMA) 2020 guideline (23). The study protocol was registered in advance with PROSPERO (registration number: CRD420261286832).

### 2.2 Inclusion and exclusion criteria

Eligible studies met the following criteria (1): observational design (cross-sectional, cohort, or case-control) (2); biochemically confirmed PA based on a standardized confirmatory test, such as the captopril challenge test, saline infusion test, fludrocortisone suppression test, or oral sodium-loading test, using criteria consistent with the 2016 Endocrine Society guideline or its 2025 update (1, 2); and (3) at least one eligible outcome reported as a mean and standard deviation or in a convertible form. For PA-versus-EH analyses, the comparator comprised adults with EH. Studies without an external EH comparator were eligible when they directly compared unilateral/lateralizing with bilateral PA. Subtype classification was based preferentially on adrenal venous sampling (AVS), surgery, or pathology; imaging-only classifications were retained for sensitivity analyses (24–26).

All control groups in the included studies consisted of adults with hypertension from whom common secondary causes (including pheochromocytoma, Cushing syndrome, renovascular hypertension, and renal parenchymal disease when specified) had been explicitly excluded or ruled out according to standard clinical practice, thus meeting the definition of EH. Three studies designated their control group as low-renin essential hypertension (LREH); for this meta-analysis, all such control groups were uniformly classified as EH and pooled accordingly. Because low-renin selection could influence electrolyte and renin-related contrasts, sensitivity analyses were repeated after excluding LREH studies, and comparator-type subgroup analyses were undertaken when sufficient studies were available.

Exclusion criteria encompassed reviews, animal experiments, case reports, and conference abstracts; studies in which the PA diagnosis was unclear or not confirmed through a standardized confirmatory test; studies for which data could not be extracted or converted; and duplicate publications (the most complete version was retained). Retrieved records were loaded into reference management software for deduplication and screening.

### 2.3 Data extraction and quality assessment

Two reviewers (Xuan Zhao and Yajing Zhao) independently screened the literature and extracted data. Extracted items included first author, publication year, country or region, study design, sample size, participant age, sex distribution, and the mean and standard deviation of each outcome. Additional study-level variables included recruitment setting, diabetes, duration of hypertension, medication preparation or washout, PA screening and confirmation procedures, subtype method, control definition, renal exclusion criteria, plasma aldosterone concentration (PAC), plasma renin activity (PRA), or direct renin concentration, ARR, urinary aldosterone, and other reported hormonal markers. For continuous variables reported as medians with interquartile ranges or ranges, means and standard deviations were estimated using the methods of Luo et al. (27) and Wan et al. (28). Values reported as mean ± standard error of the mean (SEM) were converted to standard deviations using SD = SEM × √n. The between-group difference in male proportion (PA minus EH, percentage points) was calculated for study-level sex-balance analyses.

The risk-of-bias tool was selected according to the sampling framework and longitudinal design of each study. Case-control and cohort studies were evaluated using the Newcastle–Ottawa Scale (NOS) (29). A score of ≥6/9 was used as a descriptive threshold indicating higher methodological quality. Analytical cross-sectional studies were assessed using the 2017 eight-item Joanna Briggs Institute (JBI) critical appraisal checklist (30). Each item was rated Yes, No, Unclear, or Not applicable. Item-level NOS and JBI assessments and the overall appraisal summary are provided in **Supplementary Tables S2**-**S5**. The JBI checklist does not define a validated total score or universal cutoff; therefore, no numerical summary score was calculated. Two reviewers independently assessed risk of bias, with disagreements resolved through discussion or consultation with a third reviewer when necessary.

### 2.4 Statistical analysis

Statistical analyses were performed using R software (version 4.5.1) and the metafor package (31). Because laboratory assay methods and reporting units varied across studies, the standardized mean difference (SMD; Hedges’ g with small-sample correction) was selected as the primary effect-size metric. Pooled effect sizes were estimated with the DerSimonian–Laird random-effects model (32), and 95% confidence intervals (95% CI) were computed.

Heterogeneity across studies was assessed with Cochran’s Q test and the I² statistic (33): I² < 50% indicated low, 50%–75% moderate, and above 75% substantial heterogeneity. When substantial heterogeneity was identified, its potential sources were explored through subgroup analyses and meta-regression.

Egger’s regression test (34) was used to assess publication bias, supplemented by funnel-plot inspection and the trim-and-fill method (35) as a sensitivity check. Formal testing was applied when at least 10 studies were included. Each study’s influence on the pooled effect size was examined using leave-one-out analysis to assess the robustness of the pooled estimates. In addition, radial (Galbraith) plots (36) were generated to identify outlying studies that might exert a disproportionate influence on the pooled estimate.

All eligible studies were retained in the primary pooled analyses. Because Galetta et al., 2009 reported implausibly small SDs and exerted disproportionate influence in several study-level models, *post hoc* exclusion analyses were performed as robustness checks. These analyses were not prespecified in PROSPERO, and complete-data and exclusion results are reported in parallel.

Subgroup analyses examined geographic region (East Asia vs. non-East Asia), publication period, comparator type, and, for eGFR, exclusion of preexisting renal disease. Recruitment-setting analyses were conducted for potassium and sodium, and the PA-minus-EH male-percentage difference was modeled per 10-percentage-point increment for potassium, sodium, creatinine, and eGFR. Publication year was modeled continuously. Study-level PAC was harmonized to ng/dL and examined continuously and with an exploratory 30-ng/dL cutoff. PAC, PRA, direct renin concentration, raw ARR, log-transformed ARR, and urinary aldosterone were analyzed separately by assay and scale; ARR was not derived by dividing group mean PAC by group mean renin. Hormone-outcome meta-regression was performed when at least 10 studies were available, treated as exploratory when 6–9 studies were available, and omitted when fewer than 6 were available. Urinary albumin measures were classified as urinary albumin-to-creatinine ratio/albumin-to-creatinine ratio (UACR/ACR), urinary albumin excretion, albumin concentration, or total protein/urinary protein-to-creatinine ratio (UPCR), and bicarbonate measures were separated according to direct measurement or calculation method. When units were harmonizable, parallel random-effects mean-difference (MD) analyses were performed for potassium, sodium, uric acid, and eGFR. Descriptive SMD back-conversions used the median study-specific pooled within-group SD and were not interpreted as directly pooled MDs (**Supplementary Table S6**). All tests were two-sided, with p < 0.05 denoting statistical significance.

## 3 Results

### 3.1 Literature search results

Overall, 11,473 records were retrieved from the four databases, and 9,275 remained after removal of 2,198 duplicates. Following title-and-abstract screening, 162 reports were sought for retrieval; 41 could not be retrieved, leaving 121 reports for full-text eligibility assessment. After exclusion of 47 reports, 74 studies were included in the systematic review and meta-analysis (**Figure 1**). Search strings appear in **Supplementary Table S1**.

**Figure 1:**
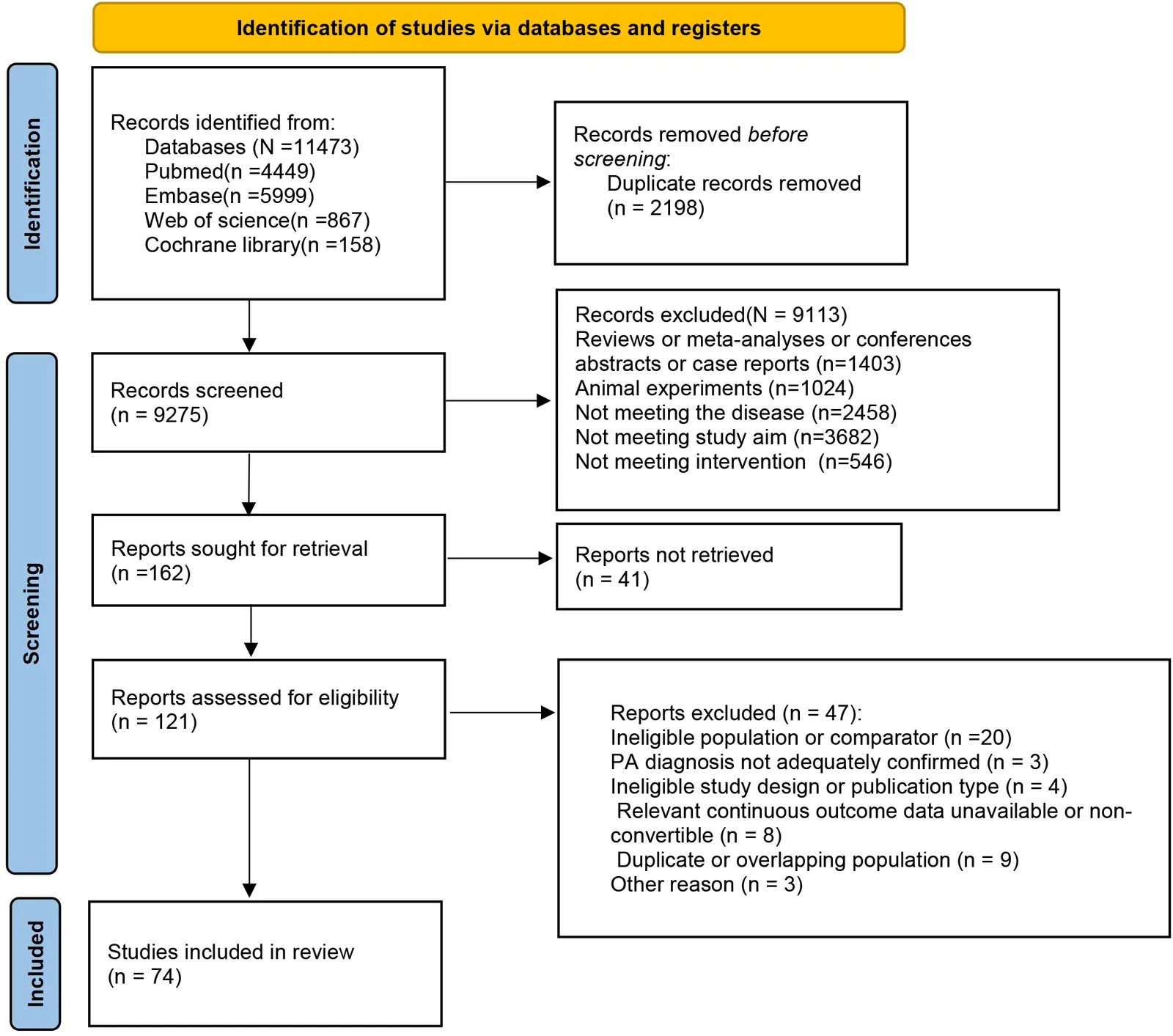
PRISMA 2020 flow diagram of study selection. The updated search identified 11,473 records. After 2,198 duplicate records were removed, 9,275 records were screened. Of 162 reports sought for retrieval, 41 were not retrieved and 121 were assessed for eligibility; 47 were excluded after full-text assessment, leaving 74 studies for inclusion. Detailed database yields and categorized full-text exclusion reasons are shown in the diagram.

### 3.2 Study characteristics and quality assessment

All 74 included studies were observational. Study characteristics are summarized in **Table 1**, including participant demographics, diabetes status, hypertension duration, medication preparation, PA screening and confirmation procedures, subtype methods, renal exclusion criteria, PAC categories, and appraisal results. Detailed item-level assessments and the overall appraisal summary are provided in **Supplementary Tables S2**-**S5**. All 54 studies assessed using the NOS met the descriptive threshold of ≥6/9. Among the 20 analytical cross-sectional studies assessed using the JBI checklist, 17 received Yes judgments for all eight items; two did not identify or address confounding factors, and one had an unclear strategy for addressing confounding. No study was excluded solely on the basis of critical appraisal. Publication years ranged from 2005 to 2026, and studies were conducted across East Asia, Europe, North Africa, and Oceania. The number of studies and sample sizes for each outcome are presented in **Table 2**.

**Table 1:** Baseline characteristics of included studies.

#	Study	Country	Study design	Recruitment setting/source	Group order	n (G1/G2)	Age, y (G1/G2)	Male, % (G1/G2)	BMI (G1/G2)	SBP/DBP, mmHg (G1/G2)	HT duration (G1/G2)	Diabetes (G1/G2)	Medication/washout	PA screening/confirmation	Subtyping	Renal disease excluded	PAC group	Appraisal result
1	Yang et al., 2017	China	Case-control (matched)	Referral/tertiary care (Single-center hypertension referral)	PA/EH	100/100	49.6 ± 12.4 / 49.9 ± 11.9	58.0 / 58.0	25.1 ± 3.4 / 25.8 ± 3.2	150 ± 21/88 ± 13 / 152 ± 19/90 ± 14	10.3 ± 7.0 y / 10.4 ± 10.0 y	17/100 / 23/100	MRA withdrawn/excluded; Diuretic management reported; Other interfering drugs withdrawn/replaced; CCB/α-blocker permitted or reported; K corrected	ARR > 24 (ng/dL)/(ng/mL/h) and PAC > 20 ng/dL; Saline infusion; post-PAC > 10 ng/dL	CT; MRI	Yes	H (≥30 ng/dL)	8/9 (NOS)
2	Zhou et al., 2021	China	Prospective cross-sectional	Referral/tertiary care (Tertiary adrenal center/CMR)	PA/EH	76/27	48 ± 11 / 46 ± 15	28.9 / 44.4	25 ± 4 / 25 ± 3	145 ± 17/93 ± 11 / 146 ± 11/94 ± 12	3.0 ± 7.2 / 6.0 ± 6.3 y	NR / NR	MRA withdrawn/excluded; Diuretic management reported; Other interfering drugs withdrawn/replaced; CCB/α-blocker permitted or reported	ARR screening per guideline; SIT and/or CCT	NR	Yes	H (≥30 ng/dL)	8/9 (NOS)
3	Zhao et al., 2025	China	Case-control	Referral/tertiary care (Hospitalized endocrine cohort)	PA/EH	152/280	51.12 ± 11.26 / 52.47 ± 15.5	38.2 / 53.2	25.47 ± 3.3 / 26.43 ± 4.0	NR / NR	7.01 ± 6.69 y / 7.08 ± 8.31 y	NR / NR	Medication/washout reported in the original study	ARR screening; SIT	AVS	No	L (<30 ng/dL)	8/9 (NOS)
4	Zhang et al., 2020	China	Case-control (matched)	Referral/tertiary care (Tertiary referral center)	PA/EH	109/109	44.9 ± 9.5 / 45.1 ± 9.7	40.4 / 40.4	22.4 ± 3.1 / 25.8 ± 3.7	157.0 ± 16.8/95.6 ± 11.6 / 156.2 ± 17.3/93.3 ± 13.8	4.6 ± 5.4 / 5.4 ± 6.0 y	NR / NR	MRA withdrawn/excluded; Diuretic management reported; Other interfering drugs withdrawn/replaced; CCB/α-blocker permitted or reported; K corrected	Standing ARR > 25; CCT; SIT if equivocal	AVS; CT; Pathology	No	H (≥30 ng/dL)	8/9 (NOS)
5	Yuan et al., 2023	China	Cross-sectional	Referral/tertiary care (Tertiary hospital)	PA/EH	62/93	54.5 ± 13.7 / 57.9 ± 14.9	56.5 / 57.0	NR / NR	NR / NR	NR / NR	NR / NR	Medication/washout reported in the original study	ARR screening; CCT/SIT	NR	No	L (<30 ng/dL)	JBI (**Supplementary Table S4**)
6	Yazidi et al., 2026	Tunisia	Retrospective cross-sectional	Referral/tertiary care (North African tertiary center)	PA/EH	112/115	50.6 ± 9.8 / 52.2 ± 21.0	54.5 / 52.2	29.4 ± 4.1 / 31.1 ± 5.7	150.7 ± 28.5/90.0 ± 15.0 / 143.5 ± 22.5/83.5 ± 7.5	NR / NR	30/112 / 31/115	NR	Center-specific diagnostic protocol; PAC > 200 pg/mL+low renin+low K, or ARR > 23; CCT/SIT	NR	No	L (<30 ng/dL)†	JBI (**Supplementary Table S4**)
7	Xu et al., 2025	China	Case-control (matched)	Referral/tertiary care (Two-center hospital cohort)	PA/EH	204/204	53.45 ± 12.70 / 53.99 ± 12.40	34.3 / 34.3	24.98 ± 3.74 / 24.79 ± 4.08	148.40 ± 19.76/89.79 ± 14.38 / 147.70 ± 22.50/90.36 ± 15.18	NR / NR	84/204 / 108/204	MRA withdrawn/excluded; Other interfering drugs withdrawn/replaced; K corrected	ARR ≥30; SIT or CCT (study-defined threshold)	NR	Yes	L (<30 ng/dL)	8/9 (NOS)
8	Xu et al., 2020 (CONPASS)	China	Prospective community-based cross-sectional	Community/primary care (large community health center)	PA/EH	40/948	45.6 ± 15.4 / 51.1 ± 12.6	47.5 / 67.1	24.3 ± 2.7 / 25.5 ± 3.3	152.4 ± 13.8/96.1 ± 8.5 / 154.1 ± 14.1/95.4 ± 9.7	NR / NR	1/40 / 21/948	NR	Community screening protocol; ARR > 20 ng/mIU and PAC > 10 ng/dL	AVS; CT	Yes	L (<30 ng/dL)	9/9 (NOS)
9	Xie et al., 2026	China	Case-control (1:1 matched)	Referral/tertiary care (Tertiary cardiovascular center)	PA/EH	138/138	51.58 ± 9.40 / 52.67 ± 10.10	57.2 / 51.4	26.63 ± 4.33 / 27.20 ± 4.02	156.83 ± 24.26/98.83 ± 15.02 / 145.02 ± 19.90/93.15 ± 13.28	NR / NR	40/138 / 22/138	MRA withdrawn/excluded; Other interfering drugs withdrawn/replaced; CCB/α-blocker permitted or reported	ARR ≥30; SIT/CCT	CT	No	NR	8/9 (NOS)
10	Wu et al., 2024	China	Cross-sectional	Referral/tertiary care (Tertiary hypertension center)	PA/EH	129/252	45.95 ± 10.54 / 44.60 ± 13.30	45.0 / 55.6	24.67 ± 3.15 / 24.94 ± 3.70	NR / NR	3.0 ± 4.8/ 2.5 ± 3.4 y	NR / NR	Medication/washout reported in the original study	ARR ≥30; SIT/CCT	NR	No	L (<30 ng/dL)	JBI (**Supplementary Table S4**)
11	Willenberg et al., 2009	Germany	Diagnostic cross-sectional	Referral/tertiary care (Two university centers)	PA/EH	35/71	57.4 ± 10.1 / 57.5 ± 12.8	48.6 / 28.2	NR / NR	154.5 ± 25.6/91.7 ± 12.9 / 150.9 ± 23.5/85.0 ± 12.6	NR / NR	NR / NR	Other interfering drugs withdrawn/replaced; CCB/α-blocker permitted or reported	ARR cutoff 33 (ng/L):(ng/L); SIT/FST	CT; MRI	No	H (≥30 ng/dL)	JBI (**Supplementary Table S4**)
12	Sun et al., 2025	China	Retrospective diagnostic cohort (PSM)	Referral/tertiary care (Tertiary cardiology center)	PA/EH	100/194	53.4 ± 11.3 / 52.8 ± 11.3	56.0 / 54.1	26.6 ± 3.6 / 26.3 ± 3.8	NR / NR	9.6 ± 9.8 y / 3.5 ± 5.8 y	NR / NR	NR	ARR ≥3.7 ng/dL per mU/L; SIT followed by CCT	AVS; CT	Yes	NR	8/9 (NOS)
13	Tang et al., 2026	China	Prospective cohort	Referral/tertiary care (West China tertiary hospital)	PA/EH	80/40	47.50 ± 10.92 / 49.68 ± 10.71	38.8 / 52.5	24.99 ± 3.54 / 25.81 ± 3.91	153.60 ± 17.55/96.86 ± 12.16 / 147.91 ± 22.36/94.28 ± 12.89	NR / NR	NR / NR	Other interfering drugs withdrawn/replaced	Repeated ARR; LC-MS/MS thresholds per study; CCT/SSST	AVS	No	L (<30 ng/dL)†	9/9 (NOS)
14	Watanabe et al., 2025	Japan	Retrospective comparative study	Referral/tertiary care (Tertiary university hospital)	PA (APA/IHA)/EH	87/99	Pooled PA not directly reported / 59 ± 13 (EH)	NR / NR	APA23.9 ± 4.0; IHA25.2 ± 2.5 / 25.9 ± 5.0	NR / NR	NR / NR	NR / NR	CCB/α-blocker permitted or reported	ARR > 200; ≥2 positive tests:CCT/FUT/SIT	AVS	No	NR	JBI (**Supplementary Table S4**)
15	Rossi et al., 2006 PAPY	Italy	Prospective multicenter cross-sectional	Referral/tertiary care (Hypertension referral centers nationwide)	PA (APA/IHA)/EH	64/426	APA51.5 ± 12; IHA49 ± 12 / 45 ± 12	NR / NR	APA28.3 ± 4.8; IHA27.0 ± 4.5 / 26.8 ± 4.8	NR/NR / 147 ± 18/95 ± 11	NR / NR	NR / NR	Untreated/newly diagnosed; CCB/α-blocker permitted or reported	ARR/PA protocol; Saline infusion	AVS; CT; MRI	Yes	L (<30 ng/dL)	JBI (**Supplementary Table S4**)
16	Qin et al., 2025	China	Retrospective comparative study	Referral/tertiary care (Tertiary hypertension center)	PA/EH	147/280	46.14 ± 10.51 / 44.57 ± 13.39	45.6 / 52.5	24.15 ± 3.39 / 24.23 ± 3.17	149.65 ± 28.67/89.92 ± 19.45 / 151.51 ± 19.51/92.07 ± 15.66	4.0 ± 4.7 y / 3.8 ± 5.2 y	21/147 / 14/280	Diuretic management reported; Other interfering drugs withdrawn/replaced; CCB/α-blocker permitted or reported	SIT/CCT	AVS; CT	No	L (<30 ng/dL)	8/9 (NOS)
17	Lv et al., 2022	China	Case-control (matched)	Referral/tertiary care (Tertiary endocrine center)	PA/EH	60/60	48.8 ± 13.1 / 48.4 ± 13.8	48.3 / 48.3	24.7 ± 3.7 / 25.3 ± 3.8	140.5 ± 13.8/86.4 ± 10.2 / 142.0 ± 13.9/86.9 ± 10.0	5.4 ± 6.5 y/ 4.1 ± 5.6 y	NR / NR	K corrected	ARR screening threshold per center; At least one of CCT/SIT/furosemide upright/oral salt loading	AVS; CT; MRI	No	H (≥30 ng/dL)	8/9 (NOS)
18	Laney et al., 2025	France	Retrospective diagnostic cohort	Referral/tertiary care (Adrenal referral center)	PA/EH	44/59	NR / NR	NR / NR	NR / NR	NR / NR	NR / NR	NR / NR	Medication/washout reported in the original study	Saline infusion test in diagnostic workup	AVS	No	L (<30 ng/dL)	JBI (**Supplementary Table S4**)
19	Kishimoto et al., 2018	Japan	Cross-sectional (matched)	Referral/tertiary care (University hypertension/vascular laboratory)	PA/EH	105/52	NR / NR	NR / NR	NR / NR	NR / NR	NR / NR	NR / NR	Other interfering drugs withdrawn/replaced	ARR screening; Confirmatory testing per Japanese guideline	AVS; CT; Surgery	No	L (<30 ng/dL)	JBI (**Supplementary Table S4**)
20	Iwakura et al., 2016	Japan	Prospective cohort	Referral/tertiary care (Tertiary PA surgical center)	PA/EH	94/100	52.0 ± 11.6 / 49.0 ± 13.0	NR / 53.0	25.1 ± 3.9 / 24.7 ± 4.0†	NR / NR	9.6 ± 7.8 / 6.8 ± 8.0 y	14/94 / 7/100	Medication/washout reported in the original study	ARR screening; Confirmatory tests per Japanese protocol	AVS; Pathology; Surgery	Yes	L (<30 ng/dL)	9/9 (NOS)
21	Huang et al., 2021	China	Case-control (matched)	Referral/tertiary care (University endocrine department)	PA/EH	174/174	46.78 ± 11.93 / 45.71 ± 13.29	47.1 / 49.4	24.28 ± 4.12 / 25.14 ± 3.80	174.41 ± 23.80/105.07 ± 14.03 / 175.91 ± 14.03/106.27 ± 14.20	4.90 ± 5.76 y / 4.42 ± 4.94 y	NR / NR	Other interfering drugs withdrawn/replaced	ARR screening; CCT/SIT and clinical criteria	AVS; CT	No	NR	8/9 (NOS)
22	Wang et al., 2019	China	Cross-sectional case-control	Referral/tertiary care (Shanghai hypertension center)	PA/EH	62/30	NR / NR	NR / NR	NR / NR	NR / NR	NR / NR	NR / NR	Other interfering drugs withdrawn/replaced	ARR ≥240 pg/mL per ng/mL/h and PAC ≥200 pg/mL; Saline infusion test	AVS; CT; Pathology	Yes	H (≥30 ng/dL)	8/9 (NOS)
23	Haze et al., 2021	Japan	Cross-sectional (single-center)	Referral/tertiary care (Cardiorenal/endocrine hospital)	PA/EH	180/66	52.7 ± 11.0 / 53.0 ± 13.2	40.0 / 62.1	25.1 ± 4.7 / 25.1 ± 4.2	NR / NR	4.7 ± 5.8 / 7.7 ± 10.5 y	18/180 / 4/66	Medication/washout reported in the original study	ARR screening; CCT/SIT and/or standard tests	AVS; CT	Yes	L (<30 ng/dL)	8/9 (NOS)
24	Chen et al., 2026	China	Retrospective diagnostic cohort	Referral/tertiary care (Tertiary cardiovascular hospital)	PA/EH	76/204	54.9 ± 10.8 / 51.6 ± 14.2	51.3 / 61.8	26.3 ± 3.6 / 27.7 ± 4.2	NR/95.97 ± 13.14 / NR/96.06 ± 15.62	NR / NR	19/76 / 30/204	Medication/washout reported in the original study	ARR/urinary biomarkers evaluated; Confirmatory testing after preparation	NR	No	L (<30 ng/dL)	7/9 (NOS)
25	Iacobellis et al., 2016	Italy	Cross-sectional case-control	Referral/tertiary care (Sapienza University Rome)	PA (APA/IHA)/EH	79/30	APA50.8 ± 8.9; IHA47.9 ± 11.6 / EH51.7 ± 13.9	50.0 / 46.7	APA28.0 ± 3.3; IHA27.9 ± 5.6 / 26.5 ± 4.2	APA149.2 ± 17.8; IHA141.1 ± 14.4/APA90.8 ± 12.7; IHA87.6 ± 9.6 / 140.6 ± 26/88 ± 11.2	NR / NR	NR / NR	NR	Established/newly diagnosed PA	AVS; Surgery	No	N/A	7/9 (NOS)
26	Moon et al., 2021	South Korea	Retrospective matched cohort (PSM)	Referral/tertiary care (Seoul National University Hospital)	PA/EH	80/80	51.7 ± 11.2 / 51.7 ± 14.4	51.2 / 53.8	25.2 ± 3.7 / 25.3 ± 3.6	NR / NR	NR / NR	16/80 / 17/80	Medication/washout reported in the original study	ARR screening; Saline infusion test	AVS	No	H (≥30 ng/dL)	8/9 (NOS)
27	Concistrè et al., 2020	Italy	Cross-sectional	Referral/tertiary care (Tertiary hypertension/endocrine center)	PA/EH	42/38	51.9 ± 11.4 / 50.3 ± 10.4	50.0 / 52.6	26.7 ± 5.4 / 25.7 ± 3.5	NR / NR	2.3 ± 1.8 y / 2.1 ± 1.7 y	NR / NR	Medication/washout reported in the original study	ARR screening; Confirmatory testing	AVS; CT	Yes	H (≥30 ng/dL)	JBI (**Supplementary Table S4**)
28	Choudhary et al., 2021	Finland	Cross-sectional (matched)	Referral/tertiary care (University hospital)	PA/EH	130/130	53.0 ± 11.4 / 52.9 ± 12.5	64.6 / 64.6	30.3 ± 5.7 / 29.5 ± 4.6	154.1 ± 17.1/91.6 ± 11.4 / 145.2 ± 20.5/90.9 ± 13.7	14.9 ± 10.3 y/11.2 ± 10.3 y	31/130 / 21/130	Medication/washout reported in the original study	ARR-based; Confirmatory workup	AVS	Yes	L (<30 ng/dL)	JBI (**Supplementary Table S4**)
29	Savard et al., 2013	France	Controlled cross-sectional study (matched)	Referral/tertiary care (Hypertension referral center)	PA/EH	459/1,290	NR / NR	NR / NR	NR / NR	NR / NR	10.9 ± 9.1 y / 8.5 ± 9.3 y	74/433 / 178/1,243	Medication/washout reported in the original study	Normal ARR required for EH; ARR screening; Confirmatory testing	AVS	No	L (<30 ng/dL)	JBI (**Supplementary Table S4**)
30	Kawashima et al., 2019	Japan	Multicenter cross-sectional (matched)	Referral/tertiary care (JPAS/JRAS centers)	PA/EH	128/128	NR / NR	NR / NR	NR / NR	NR / NR	NR / NR	NR / NR	NR	ARR screening; CCT/SIT/furosemide upright etc.	AVS; CT	Yes	L (<30 ng/dL)	8/9 (NOS)
31	Wu et al., 2011	Taiwan	Prospective multicenter cross-sectional	Referral/tertiary care (TAIPAI)	PA/EH	330/246	48.74 ± 11.66 / 50.73 ± 15.81	42.1 / 48.0	25.2 ± 3.9 / 24.9 ± 3.9	152.9 ± 22.5/93.8 ± 15.3 / 151.9 ± 20.8/89.3 ± 13.5	7.2 ± 7.2 y / 5.7 ± 6.6 y	40/330 / 18/246	Medication/washout reported in the original study	TAIPAI ARR protocol; CCT/SIT etc.	AVS; CT	No	H (≥30 ng/dL)	9/9 (NOS)
32	Kang et al., 2022	China	Retrospective cross-sectional	Referral/tertiary care (Tertiary endocrine center)	PA/EH	278/445	51 ± 12 / 51 ± 14	41.7 / 44.9	24.9 ± 4.0 / 24.7 ± 3.8†	153 ± 20/92 ± 14 / 146 ± 23/89 ± 16	7.0 ± 7.5 / 3.7 ± 5.9 y	66/278 / 124/445	Medication/washout reported in the original study	ARR > 20 pg/mL per mIU/mL; CCT, SIT or FST confirmation; EH had negative screening/confirmatory work-up and other secondary HT excluded	NR	Yes	L (<30 ng/dL)	8/9 (NOS)
33	Fogari et al., 2007	Italy	Prospective cross-sectional	Community/primary-care linked (hypertension center; primary-care referrals)	PA/EH	177/2,823	48 ± 7 / 52 ± 6	50.3 / 47.4	26.2 ± 1.9 / 26.5 ± 1.8	162 ± 3/101 ± 3 / 158 ± 6/99 ± 4	9.3 ± 3.6 / 8.1 ± 2.4	Excluded / Excluded	MRA use reported; Other interfering drugs withdrawn/replaced; CCB/α-blocker permitted or reported; K corrected	ARR > 25; IV saline suppression; dexamethasone/GRA testing; False-positive ARR/negative saline classified as EH; other secondary HT, diabetes, renal failure and obesity excluded; **Table 2**–3	CT	No	L (<30 ng/dL)	8/9 (NOS)
34	Šomlóová et al., 2010	Czech Republic	Cross-sectional case-control	Referral/tertiary care (Tertiary hypertension center)	PA/EH	100/90	49.99 ± 8.45 / 49.85 ± 11.7	57.0 / 51.1	28.72 ± 4.63 / 28.74 ± 4.63	165.86 ± 22.85/101.48 ± 14.41 / 168.39 ± 30.23/99.80 ± 19.97	10.91 ± 8.41 / 9.06 ± 9.51	20/100 / 20/90	Medication/washout reported in the original study	ARR screening; IV saline confirmation	AVS; CT; Pathology; Surgery	No	H (≥30 ng/dL)	JBI (**Supplementary Table S4**)
35	Turchi et al., 2014	Italy	Prospective cohort (baseline data)	Referral/tertiary care (Endocrine hypertension center)	PA/EH	102/132	51 ± 12 / 53 ± 9	56.9 / 43.9	26.8 ± 3.9 / 27.5 ± 3.3	160 ± 20/101 ± 12 / 150 ± 14/94 ± 7	8.3 ± 6.8 / 7.5 ± 6.9	15% / 15%	Medication/washout reported in the original study	ARR and confirmatory suppression testing	AVS; CT; Surgery	No	H (≥30 ng/dL)	8/9 (NOS)
36	Ohno et al., 2018	Japan	Retrospective multicenter case-control (matched)	Referral/tertiary care (JPAS and Kyoto control cohort)	PA/EH	236/236	58.7 ± 11.7 / 58.8 ± 11.8	47.5 / 47.5	25.1 ± 3.7 / 23.8 ± 3.5	144.7 ± 17.3/87.4 ± 12.5 / 144.7 ± 17.3/87.1 ± 12.1	5.8 ± 8.2 / 5.7 ± 7.5 y	NR / NR	Medication/washout reported in the original study	Japanese PA guideline; confirmatory testing	NR	No	L (<30 ng/dL)	8/9 (NOS)
37	Monticone et al., 2017	Italy	Prospective primary-care prevalence study	Community/primary care (Turin primary-care/referral network)	APA/BAH	27/64	NR / NR	NR / NR	NR / NR	NR / NR	NR / NR	NR / NR	NR	ARR plus confirmatory PA testing	Adrenal CT; AVS	No	H (≥30 ng/dL)	JBI (**Supplementary Table S4**)
38	Luo et al., 2016	China	Cross-sectional	Mixed/unclear (Xinjiang Hypertension Institute)	PA/non-PA HTN	216/657	47.1 ± 8.6 / 44.8 ± 9.2	57.9 / 63.0	27.4 ± 3.9 / 27.1 ± 3.7	NR / NR	6.6 ± 6.5 y / 4.3 ± 5.4 y	NR / NR	Medication/washout reported in the original study	ARR screening and confirmatory testing per protocol	AVS; CT	Yes	L (<30 ng/dL)	JBI (**Supplementary Table S4**)
39	Murata et al., 2017	Japan	Retrospective multicenter cross-sectional	Mixed (five endocrinology hospitals and community clinics)	PA (nPA/hPA)/EH	292/498	58.3 ± 13.8 / 60.9 ± 11.2	46.2 / 55.2	24.4 ± 3.8 / 24.3 ± 3.3	139.7 ± 17.6/83.7 ± 12.6 / 132.0 ± 11.9/80.0 ± 11.9	8.0 ± 10.5 y / 7.1 ± 5.2 y	75/292 (25.7%) / 158/498 (31.7%)	Other interfering drugs withdrawn/replaced	ARR plus CCT, upright furosemide and/or saline loading	AVS; CT; MRI	No	L (<30 ng/dL)	JBI (**Supplementary Table S4**)
40	Kobayashi et al., 2017	Japan	Retrospective cross-sectional	Referral/tertiary care (Nihon University)	PA/EH	73/24	48.4 ± 13.6 / 56.9 ± 21.9	47.9 / 50.0	25.0 ± 4.4 / 24.9 ± 3.2	NR / NR	NR / NR	NR / NR	Medication/washout reported in the original study	PA confirmatory testing according to Japanese criteria	AVS	No	L (<30 ng/dL)	8/9 (NOS)
41	Rosa et al., 2012	Czech Republic	Case-control (matched)	Referral/tertiary care (Tertiary hypertension center)	PA/EH	49/49	51 ± 10 / 50 ± 12	73.5 / 67.3	29.7 ± 4.3 / 30.1 ± 4.6	NR / NR	8.0 ± 9.2 y / 6.4 ± 6.9 y	NR / NR	MRA withdrawn/excluded; Other interfering drugs withdrawn/replaced; CCB/α-blocker permitted or reported	ARR ≥40 with PRA ≤0.7 ng/mL/h and PAC ≥15 ng/dL; saline infusion test (2 L over 4 h), with PA confirmed when post-infusion PAC failed to suppress below 7 ng/dL	CT	Yes	H (≥30 ng/dL)	8/9 (NOS)
42	Matsumura et al., 2006	Japan	Cross-sectional case-control	Referral/tertiary care (Kyushu University)	PA/EH	25/29	47.2 ± 11.0 / 53.3 ± 14.54	NR / NR	22.5 ± 2.5 / 22.0 ± 2.69	149.3 ± 14.5/91.8 ± 9.5 / 147.6 ± 11.31/86.6 ± 9.69	7.6 ± 6.0 y / 13.4 ± 11.31 y	NR / NR	MRA use reported	Conn adenoma established by endocrine evaluation with adrenal imaging and pathology; the original article did not restate a specific suppression-test protocol	AVS; CT; Pathology	No	H (≥30 ng/dL)	7/9 (NOS)
43	Mulatero et al., 2013	Italy	Retrospective matched cohort study	Referral/tertiary care (Tertiary hypertension referral center)	PA/EH	270/810	44 ± 8.5 / 44 ± 11.4	59.6 / 59.6	26.8 ± 4.0 / 26.7 ± 4.2	155 ± 21/96 ± 12 / 154 ± 19/95 ± 11	5.7 ± 6.0 / 6.1 ± 6.7 y	4.1% / 4.1%	Medication/washout reported in the original study	ARR and confirmatory testing according to center protocol	AVS; Pathology; Surgery	No	L (<30 ng/dL)	8/9 (NOS)
44	Muiesan et al., 2008	Italy	Case-control (matched)	Referral/tertiary care (Hypertension center)	PA/EH	125/125	50 ± 11 / 51 ± 11	56.8 / 56.8	27.4 ± 5 / 27.6 ± 5	152 ± 18/96 ± 11 / 148 ± 16/94 ± 9	NR / NR	NR / NR	Medication/washout reported in the original study	PA diagnosed by hormonal screening and confirmatory work-up; **Table 1**; overall ARR not directly reported	NR	No	NR	8/9 (NOS)
45	Jiang et al., 2016	China	Cross-sectional	Referral/tertiary care (Ruijin Hospital endocrine center)	PA/EH	242/120	49.0 ± 11.9 / 50.0 ± 12.0†	54.1 / 44.2	24.2 ± 3.1 / 25.5 ± 3.8	176.5 ± 22.4/108.2 ± 14.9 / 163.5 ± 22.5/100.0 ± 15.0	NR / NR	NR / NR	MRA use reported; Diuretic management reported; Other interfering drugs withdrawn/replaced; CCB/α-blocker permitted or reported; K corrected	2008 Endocrine Society criteria; saline infusion PAC > 10 ng/dL	AVS; CT; Pathology	No	H (≥30 ng/dL)	8/9 (NOS)
46	Galetta et al., 2009	Italy	Case-control	Referral/tertiary care (University referral center)	PA/EH	23/24	53.95 ± 8.4 / 50.63 ± 7.5	65.2 / 58.3	28.9 ± 1.6 / 26.8 ± 1.8	158.3 ± 11.3/94.1 ± 5.2 / 156.8 ± 10.8/95.1 ± 4.5	7.5 ± 2.1 y/ 7.4 ± 2.6 y	NR / NR	Medication/washout reported in the original study	Elevated ARR with confirmatory evaluation	CT; AVS	No	H (≥30 ng/dL)	8/9 (NOS)
47	Pimenta et al., 2011	Australia	Case-control (matched)	Referral/tertiary care (Endocrine Hypertension Research Centre, Brisbane)	PA/EH	21/21	55.8 ± 7.7 / 56.3 ± 10.5	71.4 / 71.4	31.7 ± 3.4 / 29.7 ± 4.5	NR / NR	NR / NR	NR / NR	Medication/washout reported in the original study	Renin-based ARR	CT; AVS	No	NR	8/9 (NOS)
48	Pilz et al., 2014	Austria	Prospective diagnostic study	Referral/tertiary care (GECOH tertiary outpatient center)	PA/EH	9/151	49.8 ± 11.6 / 51.0 ± 15.3	44.4 / 41.7	29.8 ± 7.5 / 28.6 ± 5.9	184 ± 18/109 ± 12 / 155 ± 23/94 ± 13	NR / NR	NR / NR	Medication/washout reported in the original study	AARR ≥3.7 plus post-SIT aldosterone ≥10 ng/dL; **Table 1** and first sitting measurement in **Table 2**	AVS; CT; Pathology	No	H (≥30 ng/dL)	JBI (**Supplementary Table S4**)
49	Zhang et al., 2021	China	Retrospective cross-sectional	Mixed/unclear (Xinjiang Hypertension Institute)	PA (unilateral/bilateral)/EH	169/169	48.62 ± 8.48 / 49.38 ± 8.68	56.2 / 56.2	26.55 ± 4.09 / 26.51 ± 3.90	148.38 ± 17.97/90.98 ± 14.21 / 147.39 ± 23.11/89.62 ± 15.05	Unilateral 6.6 ± 5.3; bilateral 5.1 ± 6.8 / 4.1 ± 5.2 y	30/169 / 20/169	MRA use reported; Diuretic management reported; Other interfering drugs withdrawn/replaced; CCB/α-blocker permitted or reported; K corrected	ARR > 20 and PAC > 12; saline loading with protocol adjudication	AVS	No	L (<30 ng/dL)	8/9 (NOS)
50	Yin et al., 2010	China	Cross-sectional	Referral/tertiary care (Sun Yat-sen Memorial Hospital)	PA/EH	39/296	46 ± 13 / 46 ± 13	38.5 / 48.6	24 ± 4 / 25 ± 4	152 ± 18/98 ± 12 / 146 ± 20/92 ± 14	NR / NR	Diabetes excluded / Diabetes excluded	MRA use reported; Other interfering drugs withdrawn/replaced; K corrected	Repeated upright ARR; captopril and saline confirmation	CT; Pathology; Surgery	No	H (≥30 ng/dL)	JBI (**Supplementary Table S4**)
51	Yamashita et al., 2018	Japan	Cross-sectional diagnostic study	Referral/tertiary care (JR Sapporo Hospital)	PA/EH	24/106	56 ± 12 / 54 ± 11	54.2 / 79.2	25.5 ± 3.4 / 25.1 ± 3.8	164 ± 18/94 ± 11 / 158 ± 16/95 ± 13	7/24 had duration ≥6 y / 17/106 had duration ≥6 y	NR / NR	Other interfering drugs withdrawn/replaced	ARR ≥20 and ≥1 positive challenge among saline, furosemide, captopril or ACTH	NR	No	NR	JBI (**Supplementary Table S4**)
52	Wu et al., 2011 EPC	Taiwan	Prospective multicenter cohort	Referral/tertiary care (TAIPAI tertiary multicenter cohort)	PA/EH	113/55	49.6 ± 13.0 / 50.2 ± 16.0	41.6 / 36.4	24.9 ± 4.1 / 24.3 ± 3.9	153 ± 21/92 ± 14 / 147 ± 19/87 ± 11	6.5 ± 6.3 y / 4.4 ± 6.1 y	13/113 / 4/55	Medication/washout reported in the original study	TAIPAI protocol: ARR-based screening; CCT and/or saline-loading confirmation	Clinical/imaging subtype classification	No	H (≥30 ng/dL)	7/9 (NOS)
53	van den Berg et al., 2019	Netherlands	Prospective case-control study	Referral/tertiary care (Radboud UMC and Rijnstate Hospital)	PA/EH	20/20	50.9 ± 12.2 / 48.4 ± 14.6	60.0 / 65.0	27.5 ± 5.6 / 27.4 ± 4.3	155 ± 19/91 ± 14 / 155 ± 25/91 ± 12	7.5 ± 7.9 y / 7.3 ± 6.0 y	NR / NR	Medication/washout reported in the original study	PAC/ARR screening plus saline loading; Low aldosterone and ARR criteria excluded PA in EHT	AVS	Yes	L (<30 ng/dL)	6/9 (NOS)
54	Tian et al., 2022	China	Case-control	Referral/tertiary care (Tangshan Workers Hospital)	PA/EH	91/112	51.37 ± 10.15 / 50.30 ± 11.18	42.9 / 53.6	25.71 ± 3.39 / 25.67 ± 3.10	190.80 ± 20.30/117.70 ± 12.89 / 177.01 ± 12.89/101.39 ± 9.34	5.1 ± 6.6 y / 4.8 ± 6.4 y	NR / NR	MRA use reported; Diuretic management reported; Other interfering drugs withdrawn/replaced; K corrected	Standing ARR; saline infusion or captopril confirmation	Adrenal CT; pathology in operated APA cases	Yes	L (<30 ng/dL)	8/9 (NOS)
55	Sechi et al., 2006	Italy	Prospective cohort (baseline data)	Referral/tertiary care (University of Udine hypertension unit)	PA/EH	50/100	53 ± 12 / 52 ± 9	72.0 / 72.0	28.7 ± 3.8 / 28.6 ± 2.7	166 ± 17/103 ± 9 / 167 ± 19/102 ± 9	10 ± 6 y / 10 ± 5 y	Excluded / Excluded	Medication/washout reported in the original study	PA diagnosis reported within the University of Udine prospective protocol; the accessible article did not restate the full confirmatory suppression-test details	Pathology; Surgery	No	L (<30 ng/dL)	8/9 (NOS)
56	Sang et al., 2021	China	Cross-sectional case-control	Referral/tertiary care (Nanjing endocrine hypertension center)	PA (APA/IHA)/EH	36/31	52.05 ± 12.20 / 49.29 ± 12.71	50.0 / 45.2	25.78 ± 2.82 / 26.13 ± 4.99	145.21 ± 20.82/86.78 ± 12.52 / 142.9 ± 17.74/86.19 ± 15.49	APA 8.4 ± 8.9 y; IHA 7.3 ± 8.0 y / 8.0 ± 6.2 y	3/36 / 5/31	Medication/washout reported in the original study	ARR plus saline/captopril confirmation	Clinical/imaging subtype classification	No	L (<30 ng/dL)	8/9 (NOS)
57	Ribstein et al., 2005	France	Prospective matched study	Referral/tertiary care (Montpellier university hospital)	PA/EH	25/25	49 ± 10 / 50 ± 15	68.0 / 68.0	26.8 ± 3.0 / 26.9 ± 4.5	169 ± 20/97 ± 10 / 170 ± 15/99 ± 5	9 ± 5 y / 8 ± 10 y	Excluded / Excluded	NR	Low renin/high PAC with saline non-suppression	Surgery; AVS; Pathology	No	H (≥30 ng/dL)	9/9 (NOS)
58	Lottspeich et al., 2021	Germany	Prospective matched case-control study	Referral/tertiary care (LMU tertiary center)	PA/EH	44/44	59.6 ± 12.3 / 60.6 ± 11.5	45.5 / 47.7	27.1 ± 3.9 / 26.6 ± 5.5	153.5 ± 22.2/92.0 ± 12.3 / 150.7 ± 16.9/88.9 ± 13.0	10.6 ± 9.7 / 6.1 ± 8.5 y	6/44 / 4/44	NR	ARR/AARR and confirmatory testing	NR	No	L (<30 ng/dL)	8/9 (NOS)
59	Liu et al., 2025	China	Cross-sectional (PSM)	Referral/tertiary care (Nanchang/Jiujiang hospitals)	PA/EH	546/546	48.79 ± 10.31 / 48.16 ± 10.85	56.8 / 60.3	25.94 ± 3.47 / 25.89 ± 3.78	152.51 ± 22.12/93.34 ± 15.90 / 151.81 ± 16.81/92.59 ± 12.95	4.6 ± 7.1 y / 5.3 ± 6.8 y	112/546 / 116/546	Diuretic management reported; CCB/α-blocker permitted or reported	ARR > 30 and positive confirmatory test	NR	Yes	L (<30 ng/dL)	8/9 (NOS)
60	Irita et al., 2006	Japan	Case-control (matched)	Referral/tertiary care (Ehime University)	PA/EH	15/15	47 ± 8.9 / 52 ± 5.7	40.0 / 53.3	25.4 ± 4.8 / 24.5 ± 2.8	152 ± 23/91 ± 15 / 158 ± 17/96 ± 8	NR / NR	NR / NR	Medication/washout reported in the original study	ARR, captopril non-suppression and adrenal evaluation	NR	No	L (<30 ng/dL)	7/9 (NOS)
61	Hu et al., 2015	China	Prospective cohort (pretreatment baseline data)	Referral/tertiary care (Wuhan University)	PA/LREH	25/30	45 ± 5 / 46 ± 6	44.0 / 46.7	NR / NR	152 ± 14/100 ± 8 / 154 ± 15/101 ± 9	NR / NR	NR / NR	NR	Established PA diagnosis	Surgery	No	L (<30 ng/dL)	9/9 (NOS)
62	Fallo et al., 2007	Italy	Cross-sectional (matched)	Referral/tertiary care (Padua/Turin endocrine hypertension centers)	PA/LREH	40/40	53 ± 10 / 51 ± 11	60.0 / 60.0	27.5 ± 3.9 / 26.7 ± 3.9	162 ± 22/101 ± 10 / 161 ± 18/100 ± 8	11.4 ± 9.3 y /11.3 ± 8.3 y	Excluded / Excluded	NR	Upright ARR > 40 with PAC > 15 and confirmatory work-up	AVS; CT	No	H (≥30 ng/dL)	8/9 (NOS)
63	Deng et al., 2016	China	Case-control (matched)	Referral/tertiary care (Sun Yat-sen cardiovascular center)	PA/EH	117/117	NR / NR	NR / NR	NR / NR	NR / NR	NR / NR	NR / NR	Medication/washout reported in the original study	Standard PA screening and confirmation	NR	Yes	H (≥30 ng/dL)	8/9 (NOS)
64	Steichen et al., 2011	France	Diagnostic cross-sectional	Referral/tertiary care (Tertiary hypertension unit)	PA/EH	444/2,188	51.0 ± 10.4 / 52.3 ± 13.4	66.4 / 53.1	NR / NR	149.4 ± 23.8/87.6 ± 12.6 / 149.1 ± 24.5/86.7 ± 13.4	NR / NR	NR / NR	Medication/washout reported in the original study	Guideline screening and confirmatory work-up	NR	Yes	L (<30 ng/dL)	JBI (**Supplementary Table S4**)
65	Pizzolo et al., 2017	Italy	Cross-sectional	Referral/tertiary care (Verona hypertension center)	PA/LREH	62/56	51 ± 10 / 48 ± 11	NR / NR	NR / NR	NR / NR	NR / NR	NR / NR	Medication/washout reported in the original study	ARR and intravenous salt loading; **Table 1**; K/PAC/renin/ARR are geometric means with95%CI	AVS	No	H (≥30 ng/dL)	8/9 (NOS)
66	Liu et al., 2014	China	Case-control (matched)	Referral/tertiary care (Hypertension center)	PA/EH	50/51	41.5 ± 11.4 / 42.1 ± 11.5	64.0 / 64.7	21.9 ± 3.4 / 23.1 ± 2.8	158.4 ± 14.1/96.3 ± 9.6 / 158.8 ± 10.7/98.2 ± 9.6	4.4 ± 1.3 y / 5.6 ± 1.4 y	NR / NR	Medication/washout reported in the original study	ARR plus confirmatory testing	NR	Yes	H (≥30 ng/dL)	8/9 (NOS)
67	Iacobellis et al., 2010	Italy	Cross-sectional	Referral/tertiary care (Sapienza University Rome)	PA/EH	75/192	52.77 ± 12.82 / 55.85 ± 12.09	57.3 / 53.1	27.56 ± 4.05 / 26.60 ± 3.59	140.51 ± 21.25/86.45 ± 10.93 / 140.36 ± 19.71/84.19 ± 12.04	6.5 ± 4.1 y / 7.3 ± 5.1 y	NR / NR	NR	Established PA diagnosis	Clinical/imaging subtype classification	No	H (≥30 ng/dL)	8/9 (NOS)
68	Fernández-Argüeso et al., 2021	Spain	Case-control (matched; baseline data)	Referral/tertiary care (Tertiary hospital)	PA/EH	50/50	57.4 ± 9.4 / 60.4 ± 4.5	58.0 / 56.0	30.6 ± 3.4 / 30.6 ± 3.7	143.8 ± 2.9/85.5 ± 1.5 / 139.8 ± 2.5/85.8 ± 1.2	10.7 ± 9.34 y / 6.9 ± 5.96 y	11/50 / 11/50	Medication/washout reported in the original study	ARR and confirmatory testing	AVS	No	H (≥30 ng/dL)	8/9 (NOS)
69	Catena et al., 2006	Italy	Prospective cohort (baseline data)	Referral/tertiary care (University of Udine)	PA/EH	47/274	53 ± 12 / 53 ± 12	72.3 / 70.1	28.7 ± 3.8 / 28.4 ± 2.7	166 ± 17/103 ± 10 / 167 ± 24/102 ± 12	9 ± 7 y / 9 ± 6 y	NR / NR	Medication/washout reported in the original study	ARR screening; intravenous saline-loading test	Adrenal CT; selective AVS; surgery	No	L (<30 ng/dL)	9/9 (NOS)
70	Iwakura et al., 2014	Japan	Prospective cohort (baseline subtype data)	Referral/tertiary care (Tohoku University Hospital)	APA/bilateral PA	102/111	51.0 ± 13.1 / 56.0 ± 10.5	51.0 / 33.3	24.2 ± 4.0 / 25.6 ± 4.2	154.0 ± 23.2/94.0 ± 14.1 / 148.0 ± 21.1/89.0 ± 13.7	11.0 ± 10.1 y/ 8.3 ± 9.5 y	14/102 / 19/111	MRA use reported	PA diagnosed according to the Japanese diagnostic protocol	AVS; Surgery	No	N/A	8/9 (NOS)
71	Park et al., 2017	South Korea	Retrospective multicenter cohort (baseline subtype data)	Referral/tertiary care (Three Seoul tertiary centers)	unilateral/bilateral PA	221/48	46.1 ± 11.6 / 51.9 ± 13.4	43.9 / 66.7	24.1 ± 3.7 / 25.6 ± 4.5	154.3 ± 25.6/95.4 ± 17.5 / 145.6 ± 23.3/91.6 ± 15.3	4.7 ± 5.4 / 5.5 ± 4.5 y	NR / NR	Other interfering drugs withdrawn/replaced; CCB/α-blocker permitted or reported	ARR ≥20, PAC > 15 and saline infusion	AVS; CT	No	N/A	8/9 (NOS)
72	Wu CysC 2011	Taiwan	Prospective multicenter cohort (baseline data)	Referral/tertiary care (TAIPAI: National Taiwan University Hospital and branch hospitals)	PA/EH	130/73	49.9 ± 13.4 / 51.4 ± 14.8	43.1 / 49.3	26.0 ± 4.0 / 24.7 ± 6.2	151.9 ± 21.0/90.5 ± 13.8 / 147.6 ± 19.6/85.2 ± 12.8	6.8 ± 6.9 y / 4.7 ± 6.6 y	14/130 / 6/73	MRA withdrawn/excluded	ARR; CCT and/or SIT (TAIPAI protocol)	AVS; CT; Pathology; Surgery	No	H (≥30 ng/dL)	9/9 (NOS)
73	Nakamura et al., 2021	Japan	Retrospective cross-sectional	Referral/tertiary care (Nihon University Itabashi Hospital)	PA/EH	96/17	49.7 ± 10.5 / 62.1 ± 17.8	39.6 / 52.9	25.1 ± 4.7 / 23.1 ± 4.3	137.0 ± 13.5/87.9 ± 11.3 / 148.0 ± 11.3/87.6 ± 12.9	3.4 ± 3.8 / 4.4 ± 6.5 y	NR / NR	Diuretic management reported; Other interfering drugs withdrawn/replaced; CCB/α-blocker permitted or reported	ARR screening; captopril, saline and/or furosemide-upright confirmatory testing per Japanese guideline	AVS	No	L (<30 ng/dL)	8/9 (NOS)
74	Yasuda et al., 2026	Japan	Multicenter cross-sectional registry	Referral/tertiary care (JPAS-II; 32 institutions)	PA/EH	271/171	53.9 ± 11.3 / 58.0 ± 13.4	48.3 / 41.5	NR / NR	140.4 ± 16.9/86.5 ± 13.1 / 143.5 ± 21.7/88.1 ± 14.5	NR / NR	NR / NR	MRA use reported; Diuretic management reported; Other interfering drugs withdrawn/replaced; CCB/α-blocker permitted or reported	Positive screening plus ≥1 positive/provisionally positive confirmatory test (furosemide-upright, saline infusion or captopril challenge)	AVS; AVS lateralization criteria	No	L (<30 ng/dL)	JBI (**Supplementary Table S4**)

Continuous variables are presented as mean ± SD. Values originally reported as median (interquartile range [IQR]) or median (range) were converted using the methods of Luo et al. (27) and Wan et al. (28), and values reported as mean ± SEM were converted using SD = SEM × √n. Values that could not be reliably converted because the required distributional information was unavailable were retained in their original format. Categorical variables are shown as n/N or percentages. Denominators may differ from the total sample size when data were missing.

NR, not reported; nPA, PA with PAC within the study-defined normal range; hPA, PA with high PAC; BAH, bilateral adrenal hyperplasia; LREH, low-renin essential hypertension.

Recruitment setting/source was standardized as referral/tertiary care, community/primary care, community/primary-care linked, mixed, or mixed/unclear. The community/primary-care-linked category denotes studies recruited through primary-care referrals or mixed community pathways and should not be interpreted as a purely population-based community sample.

BP, blood pressure; DBP, diastolic blood pressure; EH, essential hypertension; HT, hypertension; PA, primary aldosteronism; SBP, systolic blood pressure.

PAC group: H, PA-group mean PAC ≥30 ng/dL; L, mean PAC <30 ng/dL; NR, unavailable; N/A, not applicable to subtype-only comparisons. Study quality was assessed using the Newcastle–Ottawa Scale (NOS) for case-control and cohort studies and the eight-item Joanna Briggs Institute (JBI) checklist for analytical cross-sectional studies.

Detailed item-level risk-of-bias assessments are provided in **Supplementary Tables S2–S4**. ARR, aldosterone-to-renin ratio; AVS, adrenal venous sampling; CCT, captopril challenge test; CT, computed tomography; DRC, direct renin concentration; FST, fludrocortisone suppression test; MRA, mineralocorticoid receptor antagonist; PAC, plasma aldosterone concentration; PRA, plasma renin activity; PRC, plasma renin concentration; SIT, saline infusion test.

**Table 2:** Summary of pooled meta-analysis results across outcomes.

Outcome	k	n (PA)	n (control)	SMD (95% CI)	I² (%)	p-value	Egger p-value
Serum potassium (PA vs. hypertensive controls)	59	7,063	14,621	−1.15 (−1.27, −1.02)	91.8	< 0.001	< 0.001*
Serum potassium (unilateral/lateralizing vs. bilateral PA)	17	1,117	999	−0.85 (−1.13, −0.57)	87.8	< 0.001	NA
Serum sodium (PA vs. hypertensive controls)	31	3,380	6,062	0.51 (0.38, 0.63)	84.0	< 0.001	0.961
Serum sodium (unilateral/lateralizing vs. bilateral PA)	7	607	580	0.43 (0.22, 0.64)	66.6	< 0.001	NA
Serum creatinine (PA vs. hypertensive controls)	42	5,439	10,788	0.01 (−0.05, 0.07)	61.2	0.803	0.233
eGFR (PA vs. hypertensive controls)	36	5,307	9,991	0.02 (−0.05, 0.09)	69.4	0.625	0.223
BUN (PA vs. hypertensive controls)	8	1,271	2,392	−0.07 (−0.21, 0.06)	61.0	0.287	NA
Serum uric acid (PA vs. hypertensive controls)	23	2,857	4,293	−0.23 (−0.35, −0.12)	78.1	< 0.001	0.394
UACR/ACR (PA vs. hypertensive controls)	8	1,290	2,234	0.49 (0.33, 0.66)	72.8	< 0.001	NA
Urinary albumin excretion (PA vs. hypertensive controls)	9	576	1,102	0.73 (0.22, 1.25)	94.9	0.005	NA

k, number of studies; SMD, standardized mean difference (Hedges' g); I², heterogeneity. *Egger p-value < 0.05. The serum-potassium Egger result (p < 0.001) is from the complete 59-study dataset. NA, not assessed because k < 10 or for subtype analyses. Parallel clinical-unit mean-difference (MD) sensitivity analyses for potassium, sodium, and uric acid are shown in **Supplementary Figure S1**; descriptive SMD back-conversions are reported in **Supplementary Table S6**. PA, primary aldosteronism; eGFR, estimated glomerular filtration rate; BUN, blood urea nitrogen; UACR/ACR, urinary albumin-to-creatinine ratio/albumin-to-creatinine ratio.

### 3.3 Serum potassium

Fifty-nine studies (PA: 7,063; hypertensive controls: 14,621) were included in the comparison of serum potassium. PA patients had significantly lower serum potassium than controls (SMD = −1.15, 95% CI: −1.27 to −1.02, I² = 91.8%, p < 0.001) (**Figure 2A**). The parallel clinical-unit sensitivity analysis yielded an MD of −0.49 mmol/L (95% CI: −0.53 to −0.44) (**Supplementary Figure S1A**). In the subtype analysis, 17 studies (unilateral/lateralizing PA: 1,117; bilateral PA: 999) showed lower potassium in unilateral/lateralizing PA (SMD = −0.85, 95% CI: −1.13 to −0.57, I² = 87.8%, p < 0.001) (**Figure 2B**).

**Figure 2:**
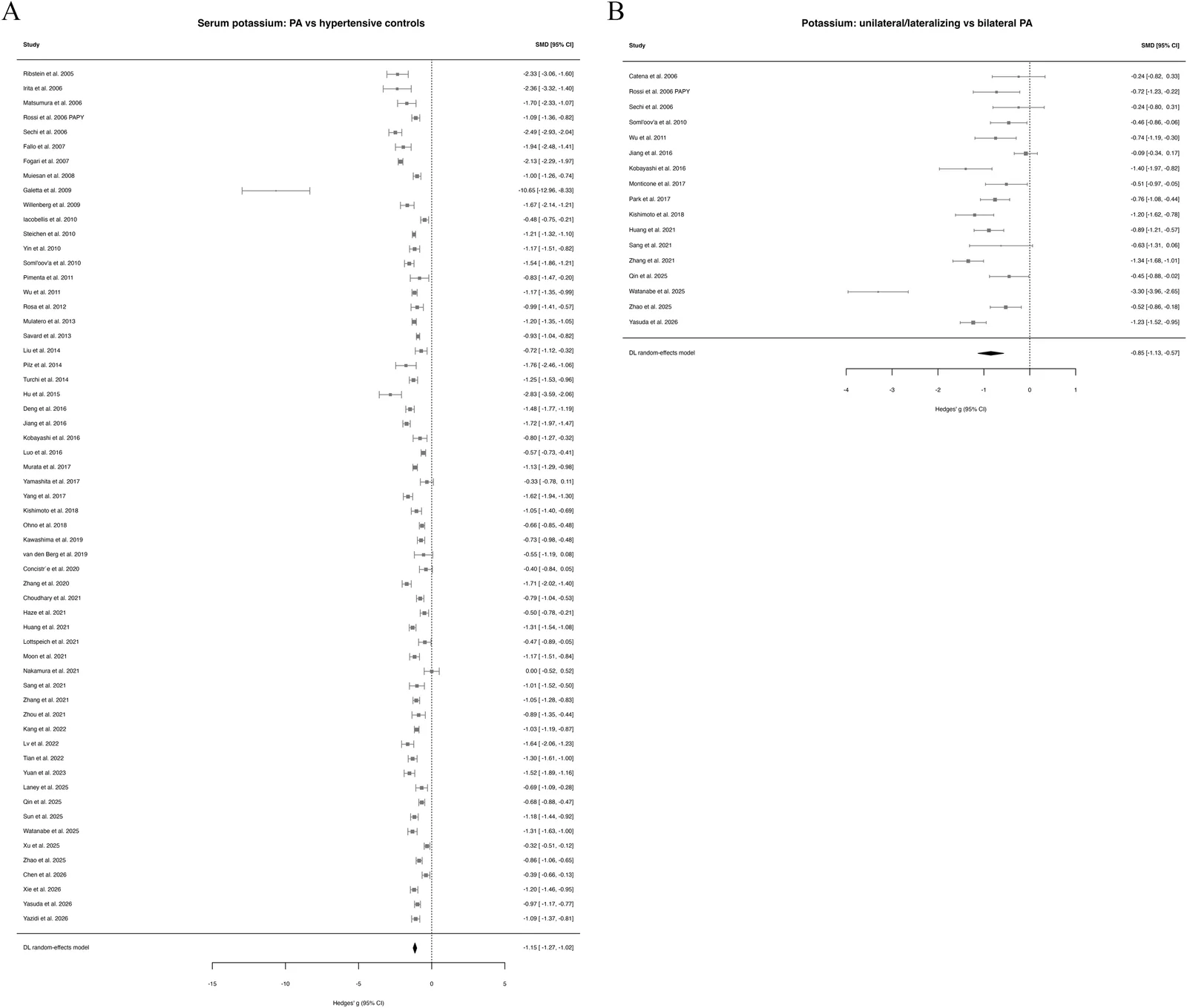
Forest plots of serum potassium. **(A)** PA versus hypertensive controls (59 studies): pooled SMD = −1.15 (95% CI: −1.27 to −1.02), I² = 91.8%, p < 0.001. **(B)** Unilateral/lateralizing PA versus bilateral PA (17 studies): pooled SMD = −0.85 (95% CI: −1.13 to −0.57), I² = 87.8%, p < 0.001.

After excluding Galetta et al., 2009, meta-regression showed that the potassium difference narrowed with advancing publication year (k = 58; slope = 0.038/year, 95% CI: 0.021 to 0.055, p < 0.001, R² = 18.2%) (**Figure 3**); the complete 59-study analysis was similar (slope = 0.042/year, R² = 16.5%) and is shown in **Supplementary Figure S2**. Studies published in or before 2015 had a larger potassium difference than those published later (SMD = −1.50 vs. −0.96; Qbetween = 17.04, p < 0.001) (**Figure 4A**), whereas East Asian and non-East Asian studies did not differ (SMD = −1.07 vs. −1.28; p = 0.148) (**Figure 4B**). Recruitment-setting subgroups also did not differ (p = 0.104), although only two community/primary-care-linked studies contributed potassium data. A 10-percentage-point increase in the PA-minus-EH male proportion was associated with a more negative potassium SMD in both the complete dataset (β = −0.274, p < 0.001) and the exclusion analysis (β = −0.241, p = 0.002) (**Supplementary Figures S3B, C**).

**Figure 3:**
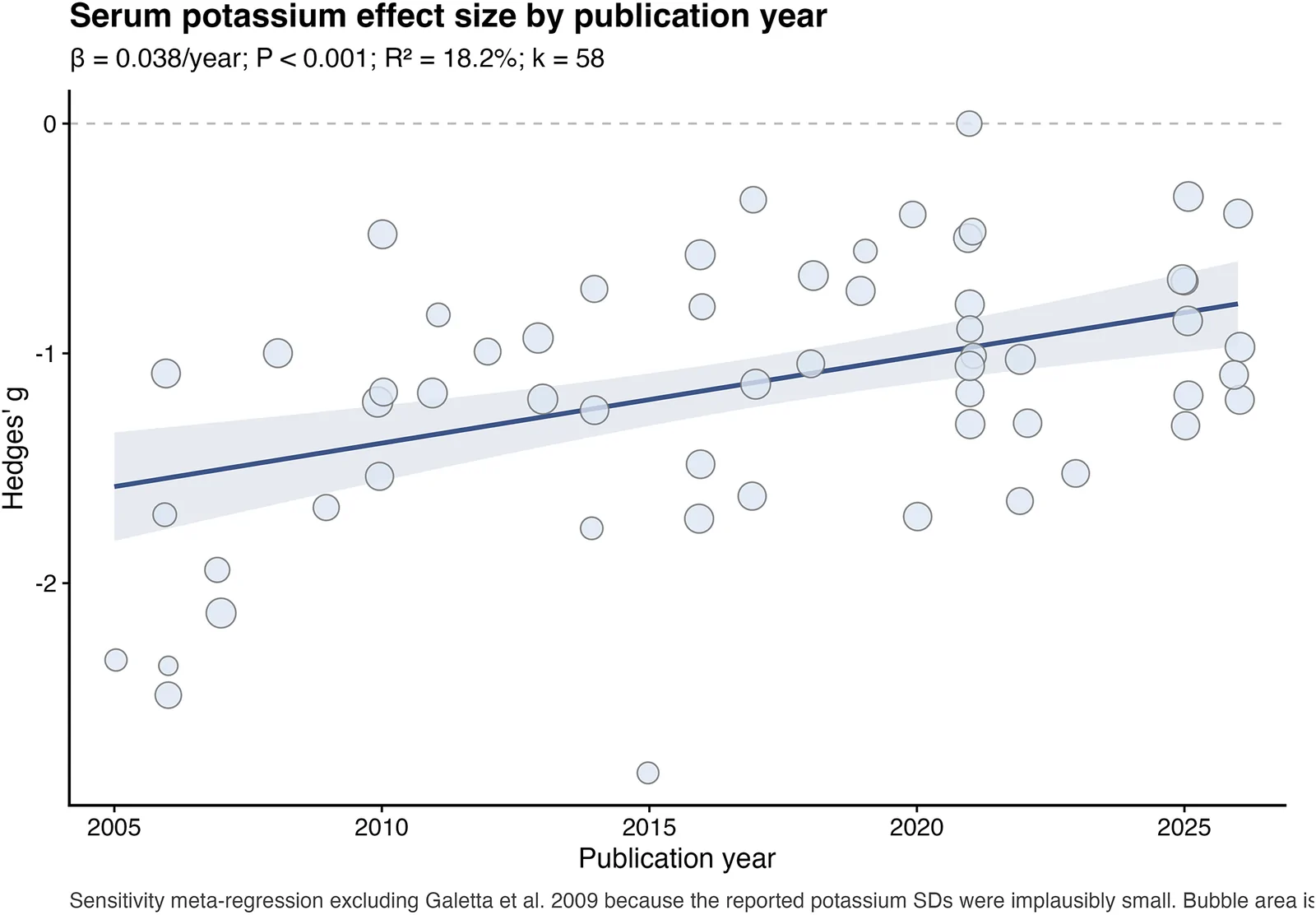
Meta-regression of serum potassium effect size on publication year. The main-text data-quality sensitivity analysis excluded Galetta et al., 2009 because of implausibly small reported SDs (k = 58). The regression coefficient was 0.038 per year (95% CI: 0.021 to 0.055, p < 0.001; R² = 18.2%), indicating attenuation of the PA–hypertensive-control potassium difference in more recent publications. The complete 59-study analysis (slope = 0.042/year; R² = 16.5%) is reported in the **Supplementary Materials**.

**Figure 4:**
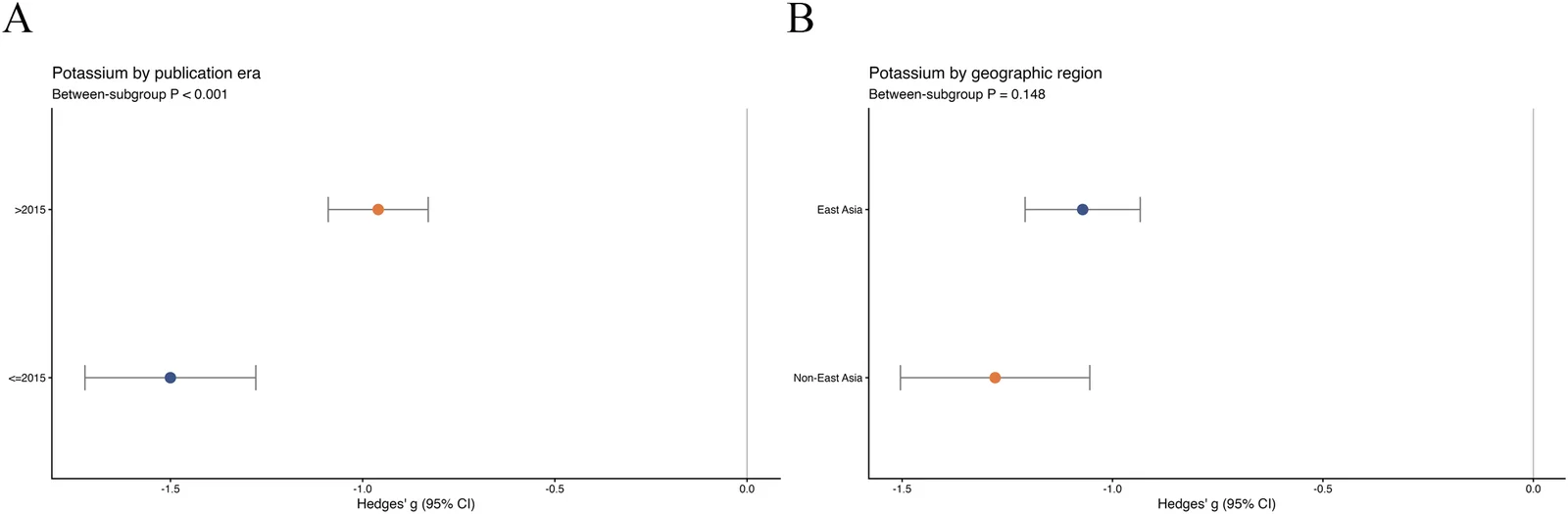
Subgroup analysis forest plots for serum potassium. **(A)** By publication period: studies published in or before 2015 (k = 23; SMD = −1.50) versus studies published after 2015 (k = 36; SMD = −0.96); between-subgroup Q = 17.04, p < 0.001. **(B)** By geographic region: East Asia (k = 36; SMD = −1.07) versus non-East Asia (k = 23; SMD = −1.28); between-subgroup Q = 2.10, p = 0.148.

### 3.4 Serum sodium

Thirty-one studies (PA: 3,380; hypertensive controls: 6,062) were included. Serum sodium concentrations were significantly elevated in PA (SMD = 0.51, 95% CI: 0.38 to 0.63, I² = 84.0%, p < 0.001) (**Figure 5A**). The parallel clinical-unit sensitivity analysis yielded an MD of +1.20 mmol/L (95% CI: +0.90 to +1.50) (**Supplementary Figure S1B**). A subtype analysis of 7 studies (unilateral/lateralizing PA: 607; bilateral PA: 580) showed higher sodium in unilateral/lateralizing PA (SMD = 0.43, 95% CI: 0.22 to 0.64, I² = 66.6%, p < 0.001) (**Figure 5B**). Publication year was not a significant moderator (slope = 0.013/year, p = 0.265). Bicarbonate evidence was limited: one study reported directly measured bicarbonate (MD = 3.00 mmol/L), two studies reported standard calculated bicarbonate (pooled MD = 0.79 mmol/L, 95% CI: −0.34 to 1.93), and one study reported potassium-adjusted calculated bicarbonate (MD = 0.85 mmol/L). These measures were not pooled together.

**Figure 5:**
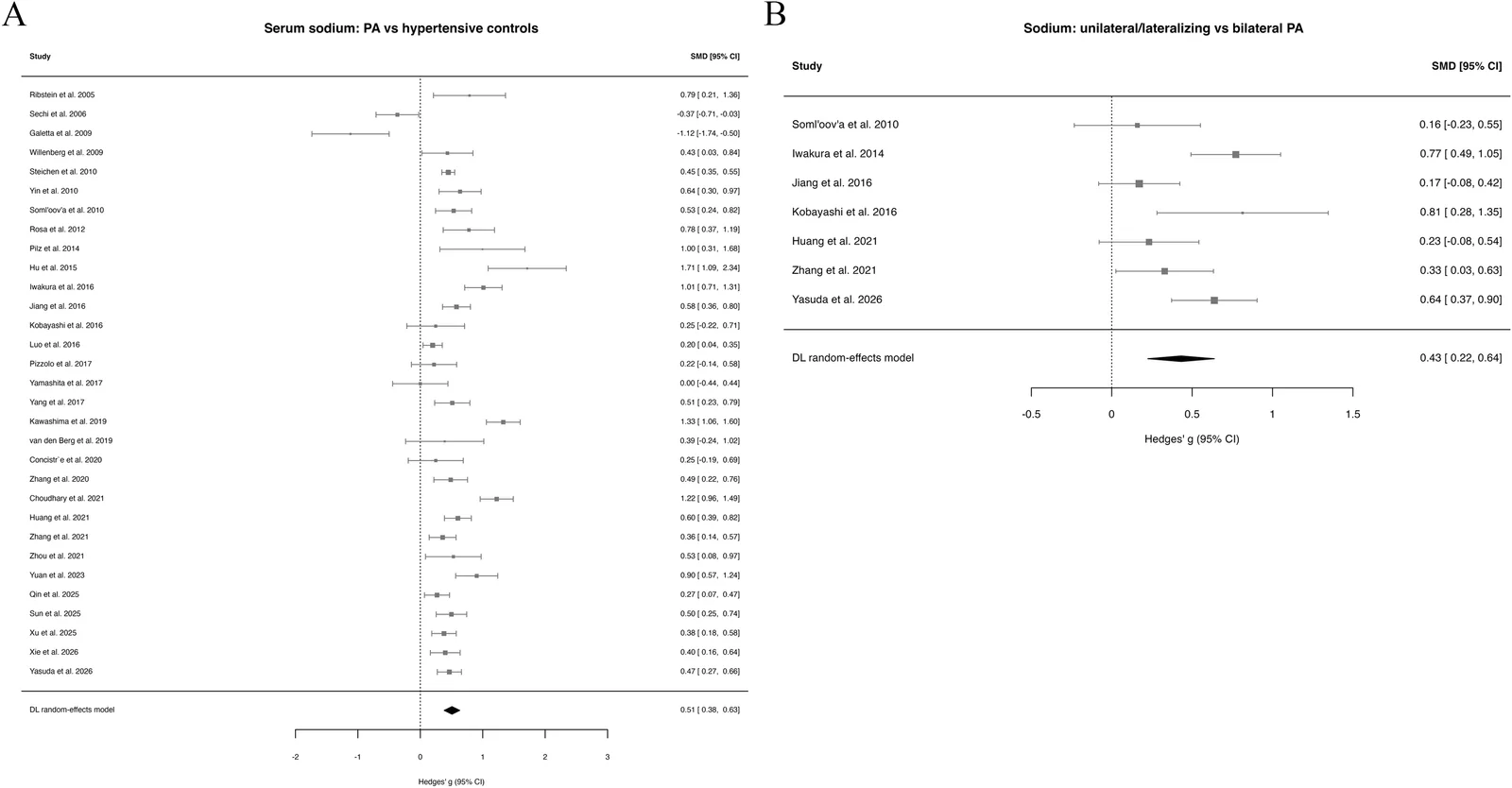
Forest plots of serum sodium. **(A)** PA versus hypertensive controls (31 studies): pooled SMD = 0.51 (95% CI: 0.38 to 0.63), I² = 84.0%, p < 0.001. **(B)** Unilateral/lateralizing PA versus bilateral PA (7 studies): pooled SMD = 0.43 (95% CI: 0.22 to 0.64), I² = 66.6%, p < 0.001.

Publication-year and geographic analyses for sodium, creatinine, eGFR, and uric acid are shown in **Supplementary Figures S4** and **S5**; the corresponding acid–base analyses are shown in **Supplementary Figures S6C–E**.

### 3.5 Serum creatinine

Forty-two studies (PA: 5,439; hypertensive controls: 10,788) were included. Serum creatinine did not differ significantly between groups (SMD = 0.01, 95% CI: −0.05 to 0.07, I² = 61.2%, p = 0.803) (**Figure 6A**). Thirteen studies comparing unilateral/lateralizing with bilateral PA also showed no significant difference (SMD = −0.07, 95% CI: −0.24 to 0.10, I² = 64.3%). Strict and expanded subtype sensitivity analyses are shown in **Supplementary Figures S7** and **S8**.

**Figure 6:**
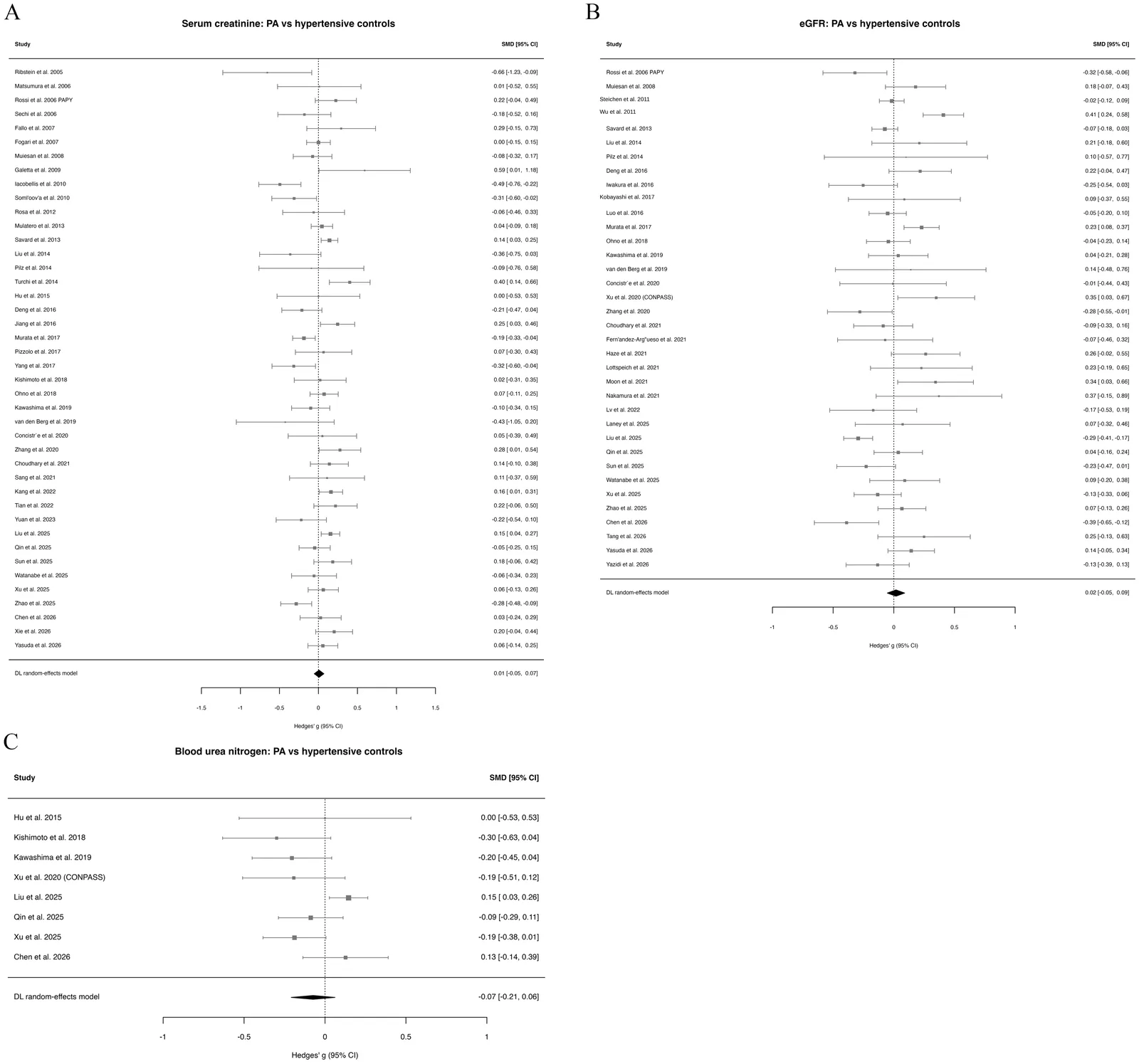
Forest plots of serum creatinine, eGFR, and BUN in PA versus hypertensive controls. **(A)** Serum creatinine (42 studies): pooled SMD = 0.01 (95% CI: −0.05 to 0.07), I² = 61.2%, p = 0.803. **(B)** eGFR (36 studies): pooled SMD = 0.02 (95% CI: −0.05 to 0.09), I² = 69.4%, p = 0.625. **(C)** BUN (8 studies): pooled SMD = −0.07 (95% CI: −0.21 to 0.06), I² = 61.0%, p = 0.287.

### 3.6 Estimated glomerular filtration rate

Thirty-six studies (PA: 5,307; hypertensive controls: 9,991) were included. Overall eGFR showed no significant difference between the two groups (SMD = 0.02, 95% CI: −0.05 to 0.09, I² = 69.4%, p = 0.625) (**Figure 6B**). The parallel MD-based analysis was also null (MD = 0.34 mL/min/1.73 m², 95% CI: −1.07 to 1.75, I² = 69.4%, p = 0.637) (**Figure 7**). In studies excluding renal disease (k = 15), the pooled SMD was −0.04 (95% CI: −0.14 to 0.06), whereas studies not explicitly excluding renal disease (k = 21) yielded 0.06 (95% CI: −0.03 to 0.16); the subgroup difference was not significant (Qbetween = 2.01, p = 0.156) (**Figure 8**). Six subtype studies showed no eGFR difference between unilateral/lateralizing and bilateral PA (SMD = 0.05, 95% CI: −0.09 to 0.19).

**Figure 7:**
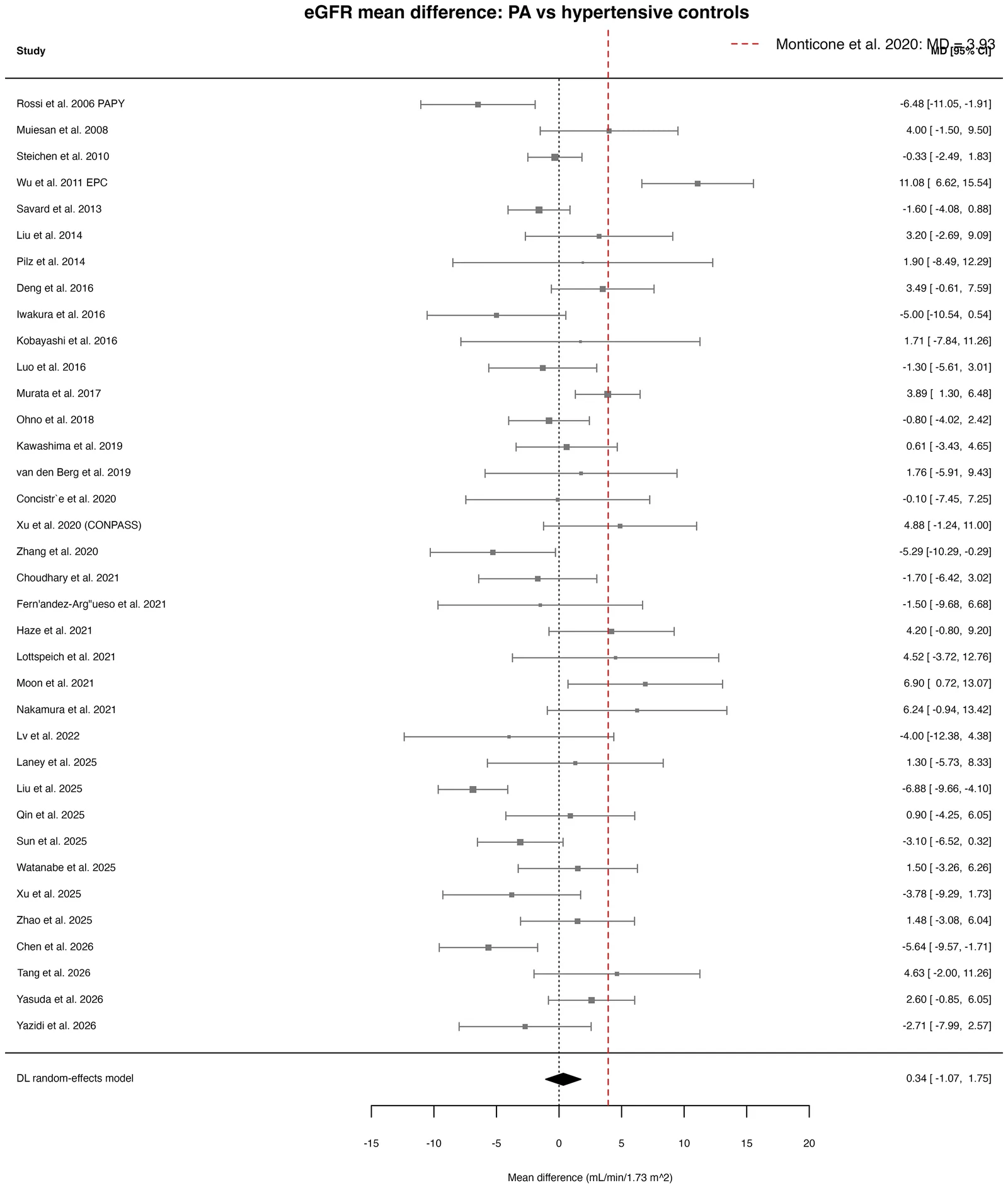
Sensitivity analysis forest plot of eGFR mean difference: PA versus hypertensive controls. Thirty-six studies were included. The pooled MD was 0.34 mL/min/1.73 m² (95% CI: −1.07 to 1.75), I² = 69.4%, p = 0.637. For comparison, the estimate of 3.93 mL/min/1.73 m² reported by Monticone et al. (2020) lies outside the 95% CI of the present analysis.

**Figure 8:**
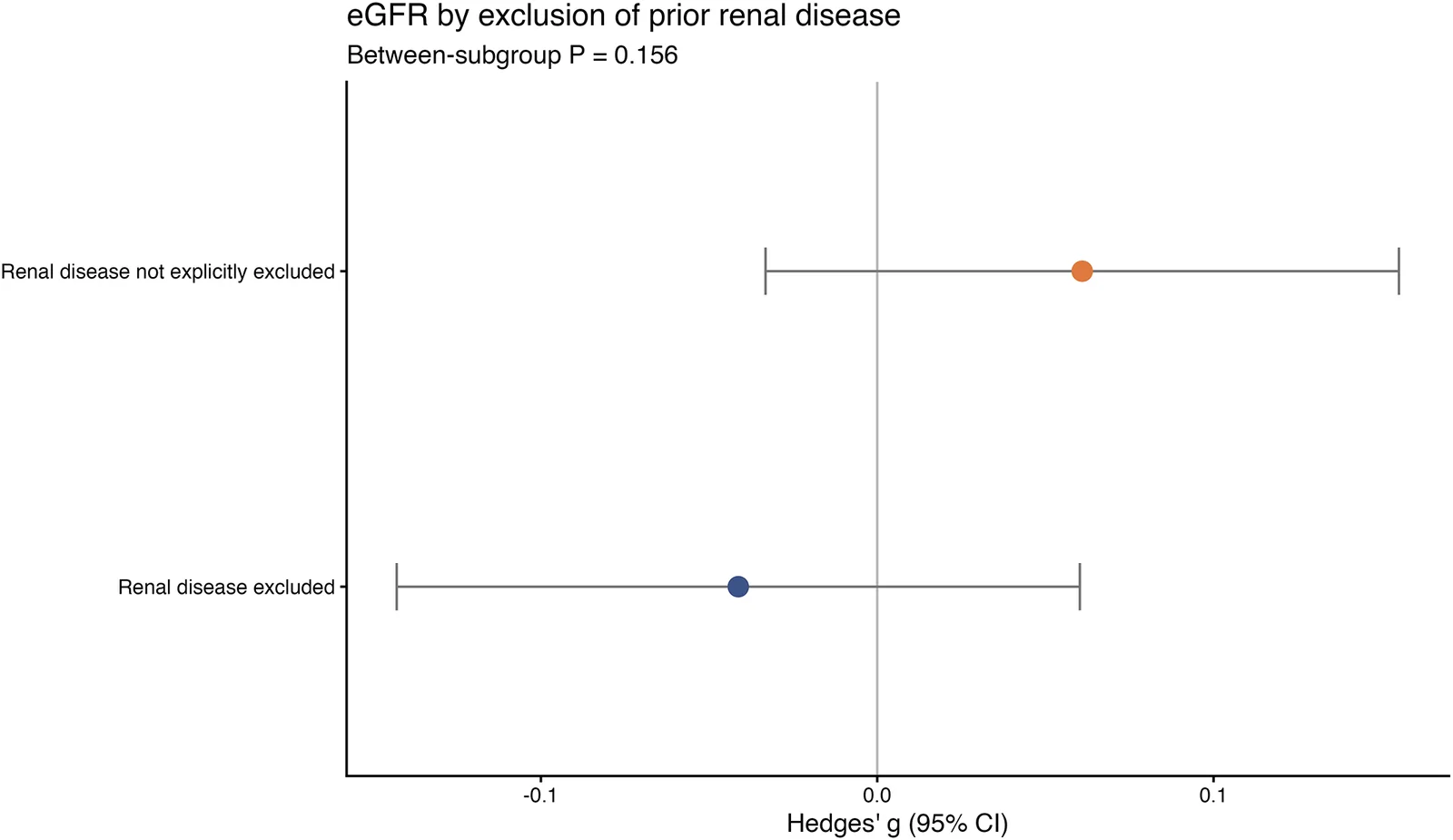
Subgroup analysis forest plot of eGFR stratified by exclusion of preexisting renal disease. Studies excluding renal disease (k = 15) yielded SMD = −0.04 (95% CI: −0.14 to 0.06), whereas studies not explicitly excluding renal disease (k = 21) yielded SMD = 0.06 (95% CI: −0.03 to 0.16). The between-subgroup difference was not significant (Q = 2.01, p = 0.156).

### 3.7 Urinary albumin outcomes

Urinary albumin outcomes were analyzed according to measurement type. UACR/ACR was higher in PA (k = 8; SMD = 0.49, 95% CI: 0.33 to 0.66, I² = 72.8%, p < 0.001) (**Figure 9A**), as was urinary albumin excretion (k = 9; SMD = 0.73, 95% CI: 0.22 to 1.25, I² = 94.9%, p = 0.005) (**Figure 9B**). A sensitivity analysis retaining one preferred albumin-specific indicator per study yielded SMD = 0.60 (k = 19; 95% CI: 0.38 to 0.82) (**Figure 9C**). Total urinary protein/UPCR was analyzed separately (k = 3; SMD = 0.17).

**Figure 9:**
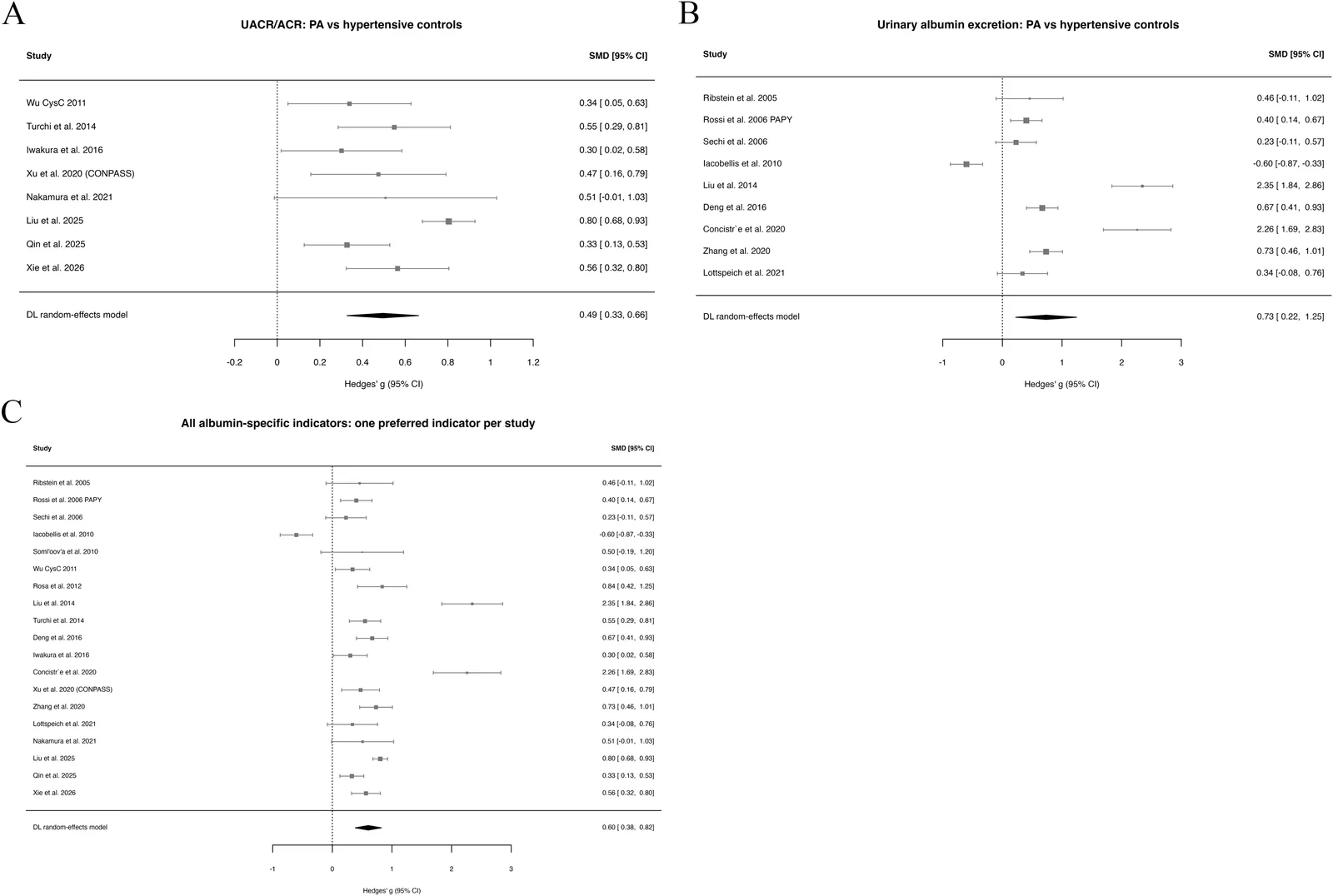
Forest plots of urinary albumin outcomes in PA versus hypertensive controls. **(A)** UACR/ACR (8 studies): pooled SMD = 0.49 (95% CI: 0.33 to 0.66), I² = 72.8%, p < 0.001. **(B)** Urinary albumin excretion (9 studies): pooled SMD = 0.73 (95% CI: 0.22 to 1.25), I² = 94.9%, p = 0.005. **(C)** Sensitivity analysis retaining one preferred albumin-specific indicator per study (19 studies): pooled SMD = 0.60 (95% CI: 0.38 to 0.82).

Additional urinary albumin and total urinary protein analyses are shown in **Supplementary Figures S6A, B**.

### 3.8 Blood urea nitrogen

Eight studies (PA: 1,271; hypertensive controls: 2,392) were included. BUN showed no significant difference between PA and controls (SMD = −0.07, 95% CI: −0.21 to 0.06, I² = 61.0%, p = 0.287) (**Figure 6C**).

### 3.9 Serum uric acid

Twenty-three studies (PA: 2,857; hypertensive controls: 4,293) were included. Serum uric acid was significantly reduced in PA (SMD = −0.23, 95% CI: −0.35 to −0.12, I² = 78.1%, p < 0.001) (**Figure 10A**). The parallel clinical-unit sensitivity analysis yielded an MD of −20.7 μmol/L (95% CI: −29.9 to −11.6) (**Supplementary Figure S1C**). The direction and significance were preserved in leave-one-out analyses. Three studies compared unilateral/lateralizing with bilateral PA, and the difference was not statistically significant (SMD = −0.19, 95% CI: −0.40 to 0.03, I² = 13.7%, p = 0.094) (**Figure 10B**).

**Figure 10:**
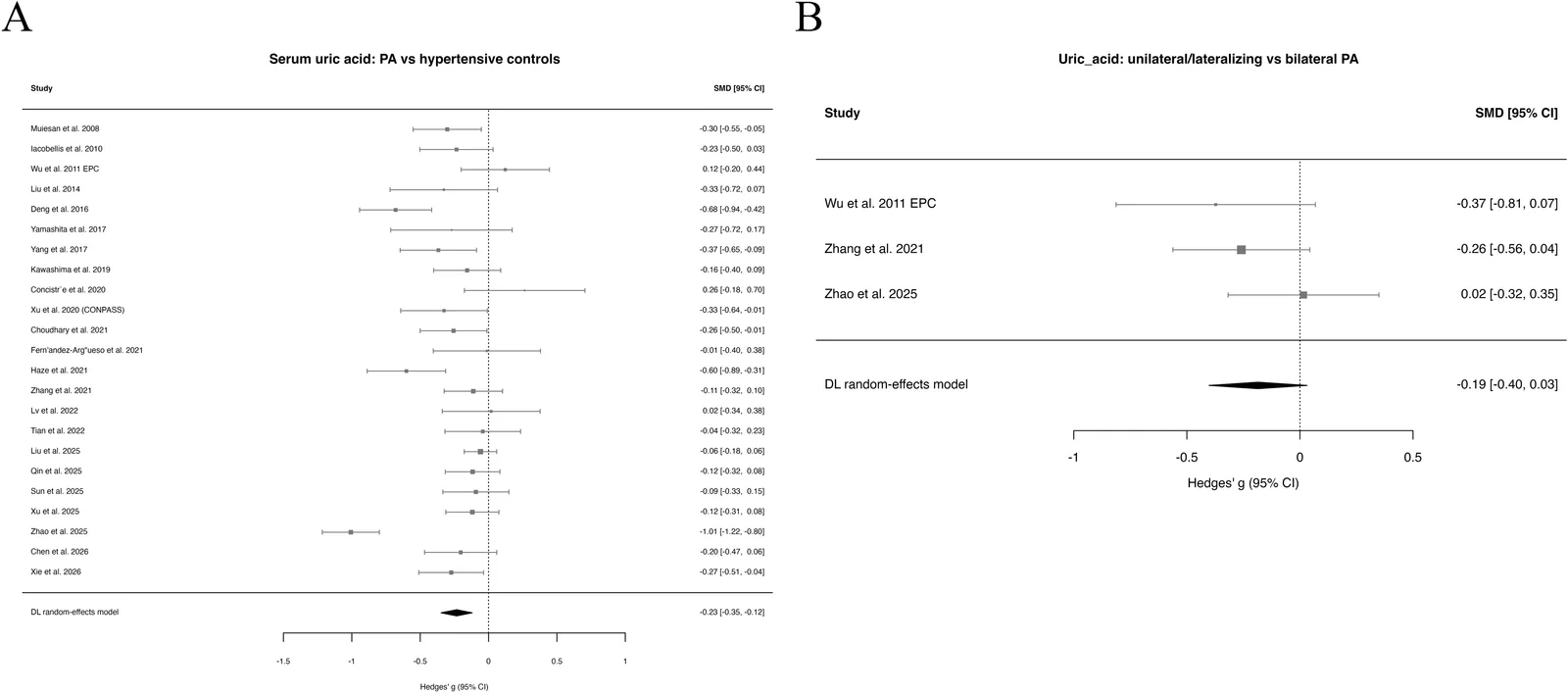
Forest plots of serum uric acid. **(A)** PA versus hypertensive controls (23 studies): pooled SMD = −0.23 (95% CI: −0.35 to −0.12), I² = 78.1%, p < 0.001. **(B)** Unilateral/lateralizing PA versus bilateral PA (3 studies): pooled SMD = −0.19 (95% CI: −0.40 to 0.03), I² = 13.7%, p = 0.094.

### 3.10 Hormone profiles and study-level meta-regression

Across compatible datasets, PA was associated with higher PAC (k = 40; SMD = 1.45), higher raw ARR (k = 27; SMD = 2.18), and higher urinary aldosterone (k = 6; SMD = 1.03), together with lower PRA (k = 26; SMD = −1.34) and lower direct renin concentration (k = 10; SMD = −0.67); 95% CIs are shown in **Supplementary Figure S9**. Original study-level hormone measurements and reported units are summarized in **Supplementary Table S7**. PRA and direct renin were analyzed separately, as were raw and log-transformed ARR. Unilateral/lateralizing PA showed higher PAC and ARR and lower PRA than bilateral PA. Detailed hormone-profile comparisons between unilateral/lateralizing and bilateral PA are shown in **Supplementary Figure S10**. Continuous PAC was not associated with potassium, sodium, or eGFR effect sizes. In complete-data potassium models, greater PRA suppression and larger ARR contrasts were associated with more negative potassium SMDs, but both associations disappeared after exclusion of Galetta et al., 2009 (**Supplementary Figures S11D–E**, **S11G–H**). For sodium, greater PRA suppression and larger ARR contrasts were associated with smaller positive sodium SMDs (**Supplementary Figures S11F**, **S11I**); ARR contrast was not associated with eGFR (p = 0.250) (**Supplementary Figure S11J**). Angiotensin I/II and catecholamines were too sparsely reported for pooling.

### 3.11 Sensitivity analysis and publication bias

Leave-one-out analyses did not materially alter the direction of the principal pooled estimates (**Supplementary Figure S12**). Excluding low-renin EH studies yielded similar estimates for potassium, sodium, creatinine, and BUN (**Supplementary Figure S13**). Comparator-type subgroups differed for potassium (p = 0.002) but not sodium or creatinine (**Supplementary Figure S14**). The cumulative analysis showed stabilization of the potassium estimate toward SMD = −1.15 (95% CI: −1.27 to −1.02) (**Figure 11**). Egger’s test was non-significant for sodium, creatinine, eGFR, and uric acid; BUN was not assessed because only eight studies were available (**Figure 12**). Funnel-plot asymmetry was present for potassium in the complete dataset (p < 0.001) and remained after excluding Galetta et al., 2009 (p = 0.007), but trim-and-fill imputed no studies, and the pooled SMD remained −1.12 (95% CI: −1.24 to −1.00) (**Figure 13**). The high-PAC subgroup had a larger potassium difference than the low-PAC subgroup (SMD = −1.44 vs. −1.03; Qbetween = 6.65, p = 0.010) (**Figure 14**). An expanded potassium analysis including studies with median or converted PAC values is shown in **Supplementary Figure S15A**; additional PAC-stratified analyses for sodium, eGFR, and UACR/ACR are presented in **Supplementary Figures S15B–D**.

**Figure 11:**
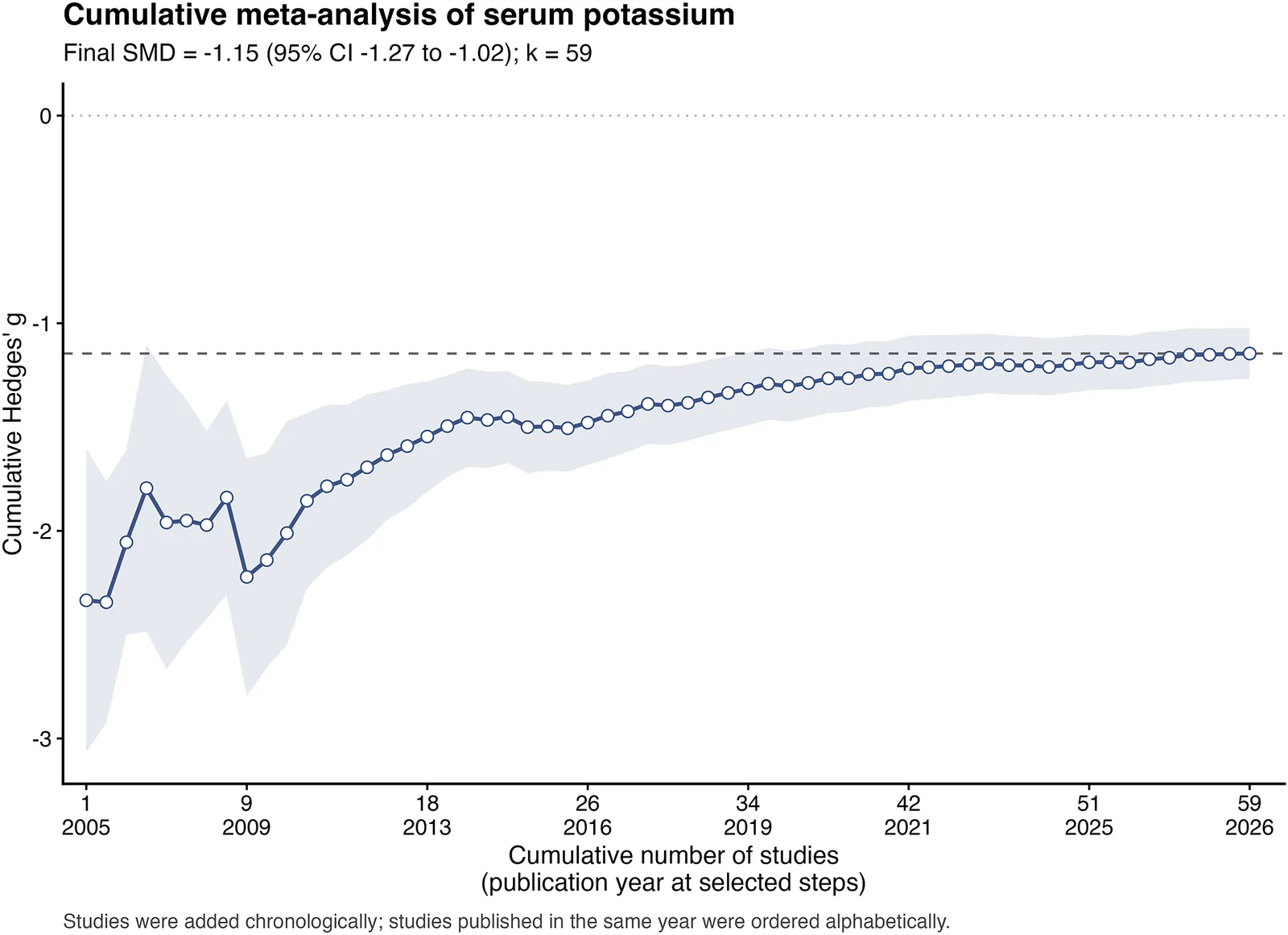
Cumulative meta-analysis of serum potassium: PA versus hypertensive controls. As studies were added chronologically, the pooled effect estimate stabilized toward the complete-analysis value of SMD = −1.15 (95% CI: −1.27 to −1.02).

**Figure 12:**
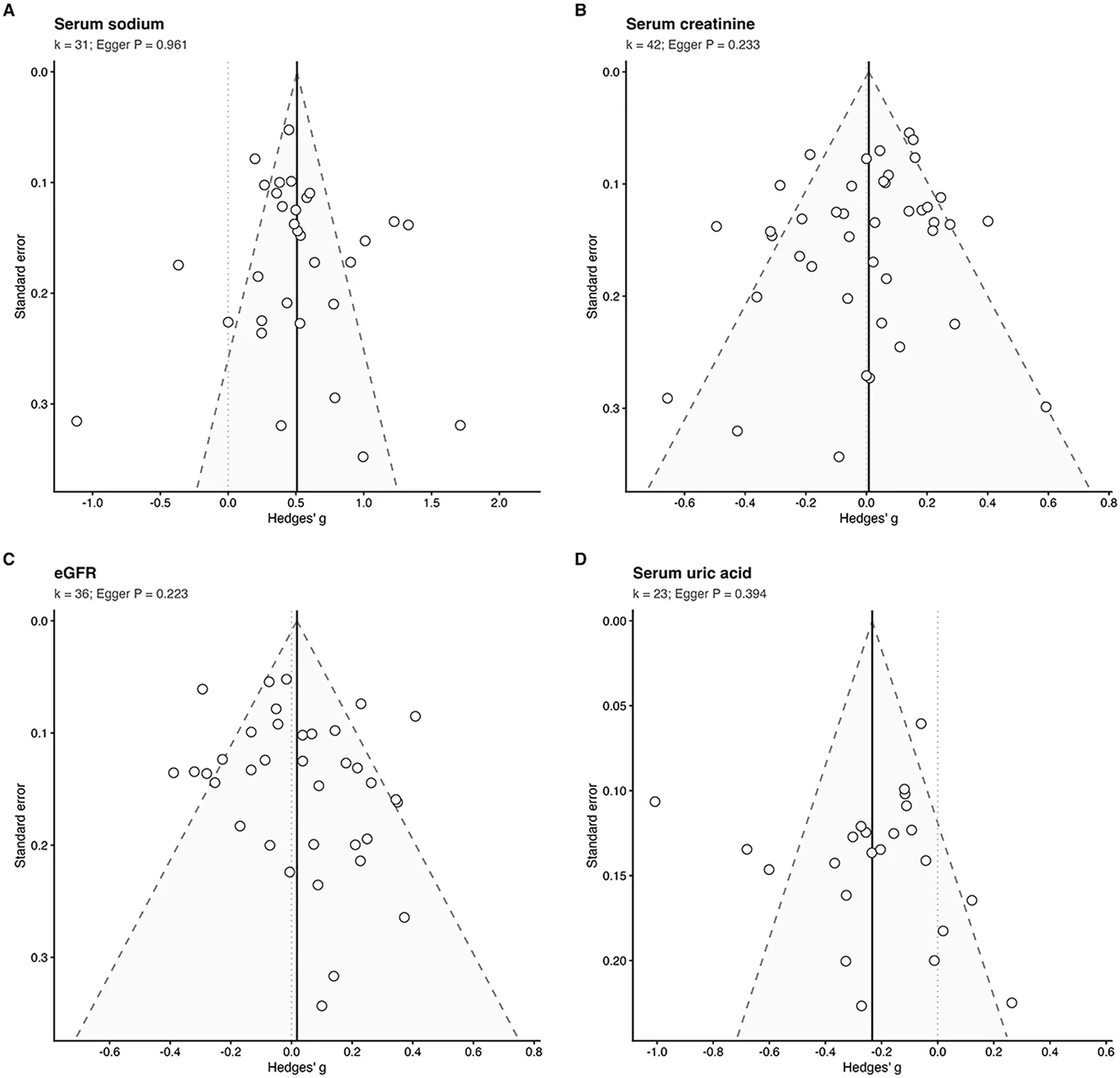
Summary funnel plots for serum sodium, serum creatinine, eGFR, and serum uric acid. Egger's regression was non-significant for serum sodium (p = 0.961), serum creatinine (p = 0.233), eGFR (p = 0.223), and serum uric acid (p = 0.394). Formal publication-bias assessment was not performed for BUN because only eight studies were available.

**Figure 13:**
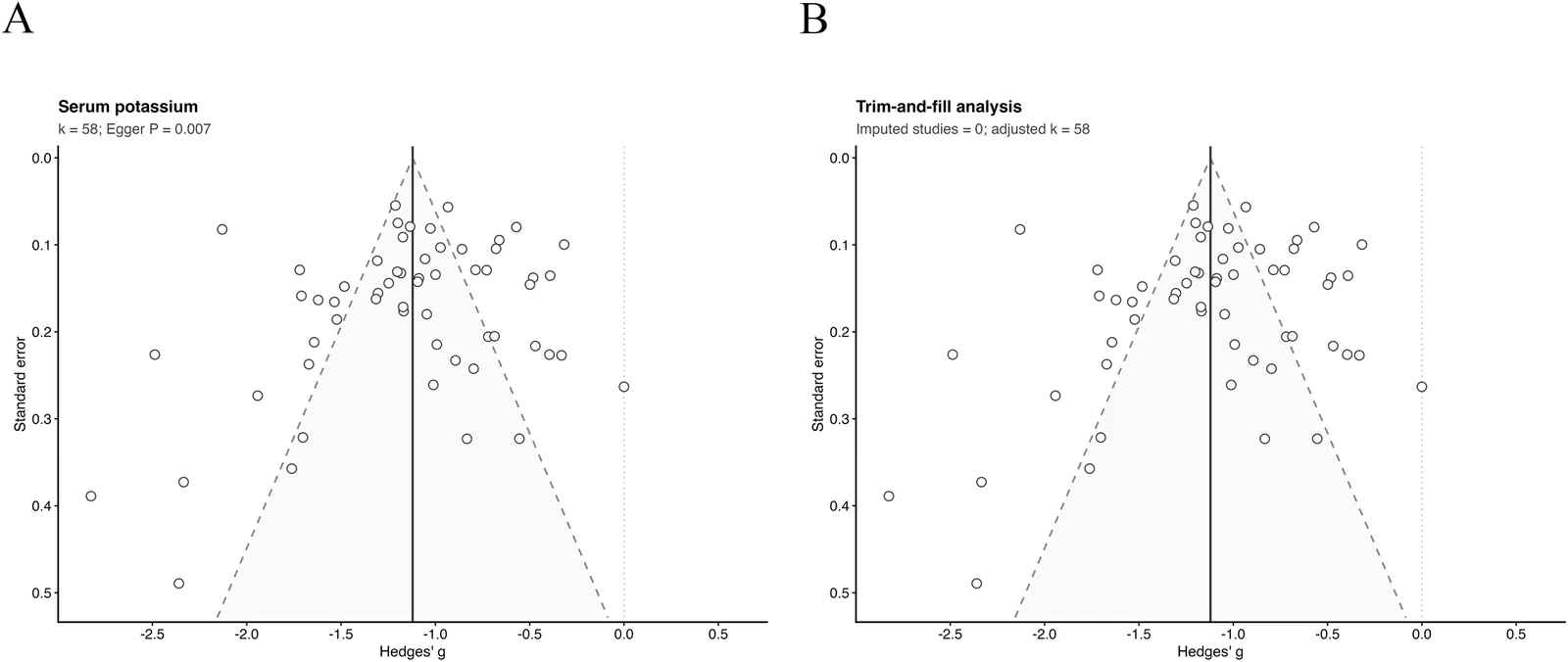
Funnel plot and trim-and-fill analysis for serum potassium: data-quality sensitivity analysis. Galetta et al., 2009 was excluded because of implausibly small reported SDs (k = 58). **(A)** Funnel-plot asymmetry persisted but was attenuated (Egger p-value = 0.007). **(B)** The trim-and-fill method imputed no studies, and the pooled SMD remained −1.12 (95% CI: −1.24 to −1.00). The corresponding complete 59-study analysis is reported separately in the **Supplementary Materials**.

**Figure 14:**
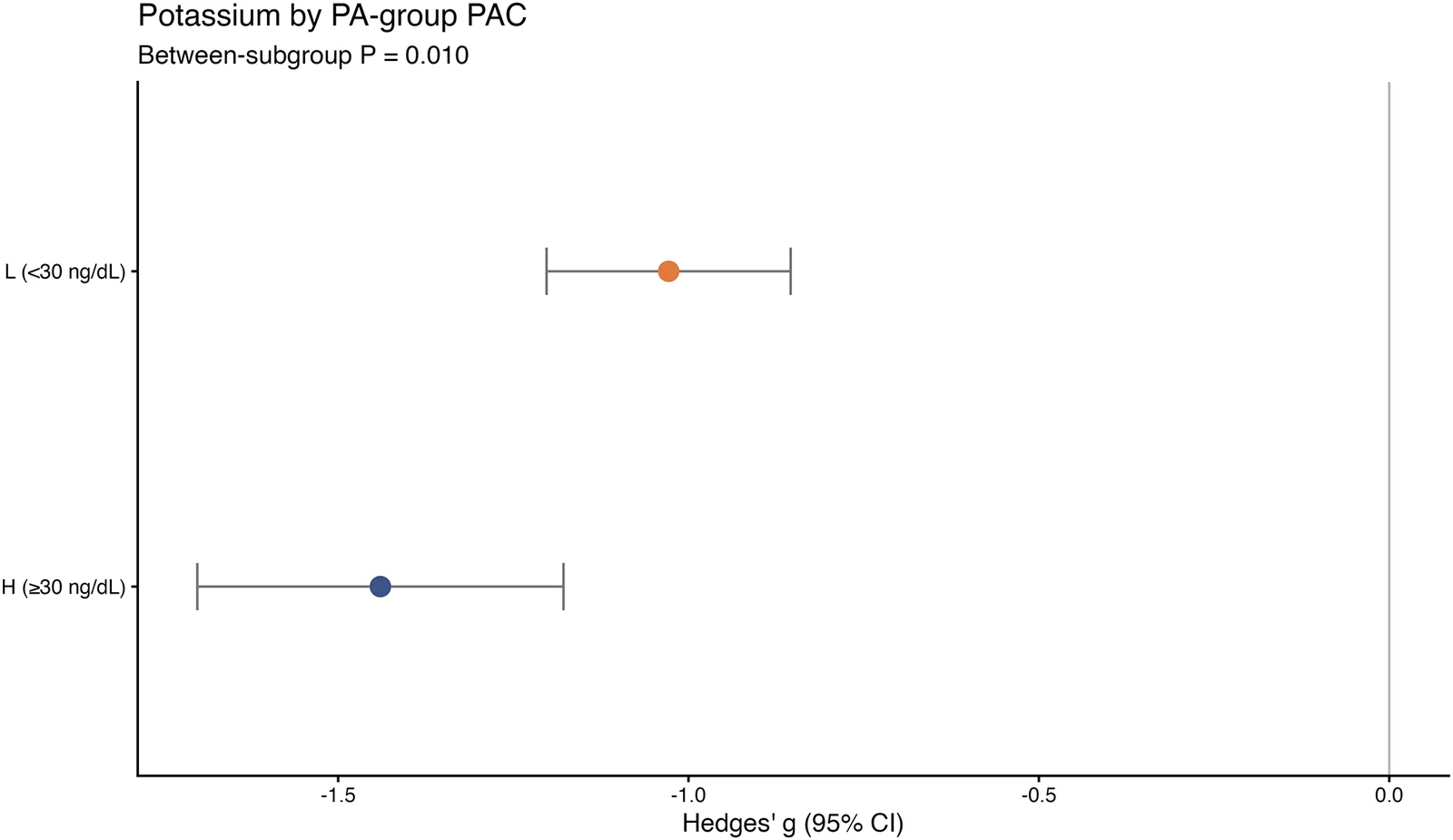
Subgroup analysis forest plot of serum potassium stratified by PA-group PAC. High-PAC studies (≥30 ng/dL; k = 19) showed a larger potassium difference than low-PAC studies (k = 28) (SMD = −1.44 vs. −1.03; Q = 6.65, p = 0.010); continuous PAC meta-regression was not significant.

## 4 Discussion

This meta-analysis of 74 studies and 26,143 participants identifies a coherent but evolving biochemical phenotype of PA. Compared with hypertensive controls, PA was associated with lower potassium, modestly higher sodium, and lower uric acid. The electrolyte contrast was greater in unilateral/lateralizing PA and in older studies, while creatinine, eGFR, and BUN were similar between groups. In contrast, UACR/ACR and urinary albumin excretion were higher in PA, revealing a separation between routine filtration markers and albuminuric renal injury.

The potassium and sodium findings are biologically consistent with mineralocorticoid receptor activation in the distal nephron, which increases epithelial sodium channel and Na+/K+-ATPase activity and promotes sodium retention and potassium secretion (9, 10). On the clinical scale, PA was associated with a mean potassium difference of −0.49 mmol/L and a sodium difference of +1.20 mmol/L. These group-average shifts are clinically meaningful without implying that every patient has hypokalemia or hypernatremia.

This distinction matters because contemporary screening increasingly identifies milder, normokalemic PA (2, 14). The larger potassium difference in studies published in or before 2015, together with attenuation across publication year, is compatible with a shift from highly selected referral phenotypes toward broader case detection. This finding supports screening strategies that do not rely on overt hypokalemia.

Unilateral/lateralizing PA showed larger potassium and sodium differences than bilateral PA, consistent with greater aldosterone autonomy in many unilateral cases (7, 37). These biochemical differences may help characterize disease severity, but treatment-directed subtyping still requires AVS and appropriate imaging (24–26).

The PAC-stratified analysis provided a similar signal: high-PAC studies showed a larger potassium contrast than low-PAC studies. Continuous PAC meta-regression was null, indicating that the categorical finding is supportive rather than evidence of a linear patient-level relationship.

The renal findings add a distinct contribution. Neither creatinine, eGFR, nor BUN differed significantly between PA and hypertensive controls, and the updated clinical-unit eGFR analysis did not reproduce the higher eGFR reported in the 2020 meta-analysis (18). The broader evidence base and inclusion of milder PA phenotypes may partly explain this difference.

A null cross-sectional eGFR result does not resolve whether renal tissue is unaffected. Aldosterone-related volume expansion and hyperfiltration can preserve or elevate filtration at diagnosis, whereas chronic mineralocorticoid excess promotes glomerular and tubulointerstitial injury (18–20). Longitudinal studies before and after targeted treatment remain necessary to separate hemodynamic from structural effects.

Against this background, the higher UACR/ACR values and urinary albumin excretion in PA are clinically informative. Albuminuria may detect glomerular or microvascular injury before a decline in creatinine-based filtration measures becomes apparent. These findings support assessment of UACR alongside creatinine and eGFR in the renal evaluation of PA.

Serum uric acid was modestly lower in PA, with a mean difference of −20.7 μmol/L. Volume expansion, hemodilution, altered renal hemodynamics, and tubular urate handling are plausible contributors (18, 21), although the available data do not distinguish among these mechanisms. Lower uric acid is therefore best viewed as part of the renal-metabolic phenotype rather than as evidence of preserved renal health.

Evidence for bicarbonate and metabolic alkalosis remained sparse and methodologically heterogeneous. Recruitment-setting analyses helped describe between-study variation but were limited by the number of studies and available community/primary-care evidence. Sex-balance and hormone-outcome analyses were further limited by assay heterogeneity and their study-level design.

The principal strength of this review is the integrated analysis of electrolytes, clinical-unit effects, PA subtypes, filtration markers, albuminuria, uric acid, and study-level hormonal profiles. Separate handling of PRA and direct renin, raw and log-transformed ARR, distinct urinary albumin measures, and directly measured versus calculated bicarbonate reduced inappropriate pooling across non-equivalent outcomes.

Several limitations remain. All included studies were observational, the majority were cross-sectional, and substantial heterogeneity persisted for several outcomes. Medication preparation, assay methods, diagnostic pathways, and case mix varied across studies, and individual-patient data were unavailable. The *post hoc* exclusion of Galetta et al., 2009 was not prespecified, although complete-data and exclusion analyses were reported in parallel.

Generalizability is greatest for referral and tertiary-care populations in East Asia and Europe, which contributed the majority of the evidence. Future studies should recruit broader primary-care populations, standardize medication preparation and biochemical measurements, and longitudinally assess UACR and filtration markers. Treatment response should also be evaluated using appropriate biomarkers during medical therapy (38) and standardized clinical and biochemical outcomes after adrenalectomy (39).

## 5 Conclusion

PA is associated with a recognizable electrolyte and renal-metabolic profile: lower potassium, modestly higher sodium, lower uric acid, and higher albuminuria than in hypertensive controls. The electrolyte contrast is more pronounced in unilateral/lateralizing PA and appears smaller in more recent studies, consistent with broader recognition of milder disease. Similar cross-sectional creatinine, eGFR, and BUN values do not exclude renal involvement; UACR may provide a more sensitive baseline signal. These findings support PA screening beyond overt hypokalemia and renal assessment that includes albuminuria alongside routine filtration markers.

## Data Availability

The original contributions presented in the study are included in the article/**Supplementary Material**. Further inquiries can be directed to the corresponding authors.
