# Supplementary material for: Biochemical and renal-metabolic profiles distinguishing primary aldosteronism from essential hypertension: a systematic review and meta-analysis with implications for endocrine hypertension screening
Source: Front Endocrinol (Lausanne). 2026 Sep 15;17:1916237. doi: 10.3389/fendo.2026.1916237 (PMC13619399; doi:10.3389/fendo.2026.1916237)
Supplement: Supplementary file 1 [file SupplementaryFile1.docx]

**Supplementary Materials**

**Table S1** Detailed Search Strategies for Each Database

*Last search date: July 13, 2026.*

**PubMed**

| # | Query |
| --- | --- |
| 1 | Hyperaldosteronism[MeSH Terms] |
| 2 | hyperaldosteronism[Title/Abstract] OR "primary hyperaldosteronism"[Title/Abstract] OR "primary aldosteronism"[Title/Abstract] OR "idiopathic aldosteronism"[Title/Abstract] OR "idiopathic hyperaldosteronism"[Title/Abstract] OR "Conn syndrome"[Title/Abstract] OR "Conn's syndrome"[Title/Abstract] OR "aldosterone-producing adenoma"[Title/Abstract] OR "aldosterone producing adenoma"[Title/Abstract] OR "unilateral primary aldosteronism"[Title/Abstract] OR "bilateral primary aldosteronism"[Title/Abstract] OR "lateralizing primary aldosteronism"[Title/Abstract] OR "lateralized primary aldosteronism"[Title/Abstract] OR "lateralised primary aldosteronism"[Title/Abstract] |
| 3 | #1 OR #2 |
| 4 | Uric Acid[MeSH Terms] |
| 5 | Potassium[MeSH Terms] |
| 6 | Sodium[MeSH Terms] |
| 7 | Blood Urea Nitrogen[MeSH Terms] |
| 8 | Glomerular Filtration Rate[MeSH Terms] |
| 9 | Creatinine[MeSH Terms] |
| 10 | Proteinuria[MeSH Terms] |
| 11 | Albuminuria[MeSH Terms] |
| 12 | Bicarbonates[MeSH Terms] |
| 13 | Alkalosis[MeSH Terms] |
| 14 | potassium[Title/Abstract] OR kalium[Title/Abstract] OR sodium[Title/Abstract] OR creatinine[Title/Abstract] OR "blood urea nitrogen"[Title/Abstract] OR BUN[Title/Abstract] OR urea[Title/Abstract] OR "glomerular filtration rate"[Title/Abstract] OR "estimated glomerular filtration rate"[Title/Abstract] OR GFR[Title/Abstract] OR eGFR[Title/Abstract] OR "uric acid"[Title/Abstract] OR urate[Title/Abstract] OR proteinuria[Title/Abstract] OR albuminuria[Title/Abstract] OR microalbuminuria[Title/Abstract] OR "urinary albumin"[Title/Abstract] OR "urinary albumin excretion"[Title/Abstract] OR "albumin excretion rate"[Title/Abstract] OR UACR[Title/Abstract] OR UAER[Title/Abstract] OR "urinary albumin-to-creatinine ratio"[Title/Abstract] OR "albumin-to-creatinine ratio"[Title/Abstract] OR bicarbonate[Title/Abstract] OR bicarbonates[Title/Abstract] OR HCO3[Title/Abstract] OR "total CO2"[Title/Abstract] OR "total carbon dioxide"[Title/Abstract] OR alkalosis[Title/Abstract] OR "metabolic alkalosis"[Title/Abstract] |
| 15 | #3 AND (#4 OR #5 OR #6 OR #7 OR #8 OR #9 OR #10 OR #11 OR #12 OR #13 OR #14) |

**Embase**

| **#** | **Query** |
| --- | --- |
| 1 | 'primary hyperaldosteronism'/exp |
| 2 | hyperaldosteronism:ti,ab,kw OR 'primary hyperaldosteronism':ti,ab,kw OR 'primary aldosteronism':ti,ab,kw OR 'aldosteronism primary':ti,ab,kw OR aldosteronism:ti,ab,kw OR 'idiopathic aldosteronism':ti,ab,kw OR 'idiopathic hyperaldosteronism':ti,ab,kw OR 'conn syndrome':ti,ab,kw OR 'conn s syndrome':ti,ab,kw OR 'conns syndrome':ti,ab,kw OR 'aldosterone producing adenoma':ti,ab,kw OR 'aldosterone-producing adenoma':ti,ab,kw OR 'unilateral primary aldosteronism':ti,ab,kw OR 'bilateral primary aldosteronism':ti,ab,kw OR 'lateralizing primary aldosteronism':ti,ab,kw OR 'lateralized primary aldosteronism':ti,ab,kw OR 'lateralised primary aldosteronism':ti,ab,kw |
| 3 | #1 OR #2 |
| 4 | 'potassium'/exp |
| 5 | 'sodium'/exp |
| 6 | 'creatinine'/exp |
| 7 | 'urea nitrogen blood level'/exp |
| 8 | 'glomerulus filtration rate'/exp |
| 9 | 'uric acid'/exp |
| 10 | 'albuminuria'/exp |
| 11 | 'proteinuria'/de |
| 12 | 'bicarbonate'/exp |
| 13 | 'alkalosis'/exp |
| 14 | potassium:ti,ab,kw OR kalium:ti,ab,kw OR sodium:ti,ab,kw OR natrium:ti,ab,kw OR creatinine:ti,ab,kw OR creatinin:ti,ab,kw OR 'serum creatinine':ti,ab,kw OR 'glomerular filtration rate':ti,ab,kw OR 'estimated glomerular filtration rate':ti,ab,kw OR egfr:ti,ab,kw OR gfr:ti,ab,kw OR 'renal function':ti,ab,kw OR 'kidney function':ti,ab,kw OR 'renal impairment':ti,ab,kw OR 'kidney impairment':ti,ab,kw OR 'blood urea nitrogen':ti,ab,kw OR bun:ti,ab,kw OR 'plasma urea nitrogen':ti,ab,kw OR 'serum urea nitrogen':ti,ab,kw OR 'urea nitrogen blood level':ti,ab,kw OR 'serum urea':ti,ab,kw OR 'uric acid':ti,ab,kw OR urate:ti,ab,kw |
| 15 | albuminuria:ti,ab,kw OR microalbuminuria:ti,ab,kw OR 'urinary albumin':ti,ab,kw OR 'urine albumin':ti,ab,kw OR 'urinary albumin excretion':ti,ab,kw OR 'urinary albumin excretion rate':ti,ab,kw OR 'albumin excretion rate':ti,ab,kw OR uacr:ti,ab,kw OR uaer:ti,ab,kw OR 'urinary albumin to creatinine ratio':ti,ab,kw OR 'urine albumin to creatinine ratio':ti,ab,kw OR 'albumin to creatinine ratio':ti,ab,kw OR 'albumin creatinine ratio':ti,ab,kw OR proteinuria:ti,ab,kw OR 'urinary protein':ti,ab,kw OR 'urinary protein excretion':ti,ab,kw |
| 16 | bicarbonate:ti,ab,kw OR bicarbonates:ti,ab,kw OR hco3:ti,ab,kw OR 'serum bicarbonate':ti,ab,kw OR 'total co2':ti,ab,kw OR 'total carbon dioxide':ti,ab,kw OR 'serum carbon dioxide':ti,ab,kw OR 'metabolic alkalosis':ti,ab,kw OR alkalosis:ti,ab,kw OR 'base excess':ti,ab,kw |
| 17 | #4 OR #5 OR #6 OR #7 OR #8 OR #9 OR #10 OR #11 OR #12 OR #13 OR #14 OR #15 OR #16 |
| 18 | #3 AND #17 |

**Cochrane Library**

| **#** | **Query** |
| --- | --- |
| 1 | MeSH descriptor: [Hyperaldosteronism] explode all trees |
| 2 | (hyperaldosteronism OR "primary hyperaldosteronism" OR "primary aldosteronism" OR "idiopathic aldosteronism" OR "idiopathic hyperaldosteronism" OR "Conn syndrome" OR "Conn's syndrome" OR "aldosterone-producing adenoma" OR "aldosterone producing adenoma" OR "unilateral primary aldosteronism" OR "bilateral primary aldosteronism" OR "lateralizing primary aldosteronism" OR "lateralized primary aldosteronism" OR "lateralised primary aldosteronism"):ti,ab,kw |
| 3 | #1 OR #2 |
| 4 | MeSH descriptor: [Uric Acid] explode all trees |
| 5 | MeSH descriptor: [Potassium] explode all trees |
| 6 | MeSH descriptor: [Sodium] explode all trees |
| 7 | MeSH descriptor: [Blood Urea Nitrogen] explode all trees |
| 8 | MeSH descriptor: [Glomerular Filtration Rate] explode all trees |
| 9 | MeSH descriptor: [Creatinine] explode all trees |
| 10 | MeSH descriptor: [Proteinuria] explode all trees |
| 11 | MeSH descriptor: [Albuminuria] explode all trees |
| 12 | MeSH descriptor: [Bicarbonates] explode all trees |
| 13 | MeSH descriptor: [Alkalosis] explode all trees |
| 14 | (potassium OR kalium OR sodium OR creatinine OR "blood urea nitrogen" OR BUN OR urea OR "glomerular filtration rate" OR "estimated glomerular filtration rate" OR GFR OR eGFR OR "renal function" OR "kidney function" OR "renal impairment" OR "kidney impairment" OR "renal injury" OR "kidney injury" OR "renal damage" OR "kidney damage" OR "uric acid" OR urate OR proteinuria OR albuminuria OR microalbuminuria OR "urinary albumin" OR "urine albumin" OR "urinary albumin excretion" OR "albumin excretion rate" OR UACR OR UAER OR "urinary albumin-to-creatinine ratio" OR "urine albumin-to-creatinine ratio" OR "albumin-to-creatinine ratio" OR "urinary protein" OR "urine protein" OR bicarbonate OR bicarbonates OR HCO3 OR "serum bicarbonate" OR "plasma bicarbonate" OR "total CO2" OR "total carbon dioxide" OR alkalosis OR "metabolic alkalosis"):ti,ab,kw |
| 15 | #4 OR #5 OR #6 OR #7 OR #8 OR #9 OR #10 OR #11 OR #12 OR #13 OR #14 |
| 16 | #3 AND #15 |

**Web of Science**

| **#** | **Query** |
| --- | --- |
| 1 | TS=(Hyperaldosteronism OR Aldosteronism OR "primary hyperaldosteronism" OR "primary aldosteronism" OR "aldosteronism primary" OR "idiopathic aldosteronism" OR "idiopathic hyperaldosteronism" OR "Conn syndrome" OR "Conn's syndrome" OR "Conns syndrome" OR "aldosterone-producing adenoma" OR "aldosterone producing adenoma" OR "unilateral primary aldosteronism" OR "bilateral primary aldosteronism" OR "lateralizing primary aldosteronism" OR "lateralized primary aldosteronism" OR "lateralised primary aldosteronism") |
| 2 | TS=(potassium OR kalium OR sodium OR natrium OR creatinine OR "serum creatinine" OR "glomerular filtration rate" OR "estimated glomerular filtration rate" OR eGFR OR GFR OR "renal function" OR "kidney function" OR "renal impairment" OR "kidney impairment" OR "blood urea nitrogen" OR BUN OR "plasma urea nitrogen" OR "serum urea nitrogen" OR "serum urea" OR "uric acid" OR urate OR albuminuria OR microalbuminuria OR "urinary albumin" OR "urine albumin" OR "urinary albumin excretion" OR "urinary albumin excretion rate" OR "albumin excretion rate" OR UACR OR UAER OR "urinary albumin-to-creatinine ratio" OR "urine albumin-to-creatinine ratio" OR "albumin-to-creatinine ratio" OR "albumin creatinine ratio" OR proteinuria OR "urinary protein" OR "urinary protein excretion" OR bicarbonate OR bicarbonates OR HCO3 OR "serum bicarbonate" OR "total CO2" OR "total carbon dioxide" OR "serum carbon dioxide" OR "metabolic alkalosis" OR alkalosis OR "base excess") |
| 3 | #1 AND #2 |

References of Included Studies

1. Yang Y, Zhu LM, Xu JZ, et al. Comparison of left ventricular structure and function in primary aldosteronism and essential hypertension by echocardiography. Hypertens Res. 2017;40:243-250. doi:10.1038/hr.2016.127.

2. Zhou F, Wu T, Wang W, et al. CMR-verified myocardial fibrosis is associated with subclinical diastolic dysfunction in primary aldosteronism patients. Front Endocrinol (Lausanne). 2021;12:672557. doi:10.3389/fendo.2021.672557.

3. Zhao X, Jiao H, Zhang R, et al. Association between serum uric acid levels and primary aldosteronism. BMC Nephrol. 2025;26:618. doi:10.1186/s12882-025-04504-6.

4. Zhang SL, Gao JW, Guo Y, et al. Associations between metabolic profiles and target-organ damage in Chinese individuals with primary aldosteronism. Front Endocrinol (Lausanne). 2020;11:547356. doi:10.3389/fendo.2020.547356.

5. Yuan H, Wang B, Yang Y, Li X. Diagnostic value of the fractional excretion of urine potassium for primary aldosteronism. Ann Transl Med. 2023;11:120. doi:10.21037/atm-22-6410.

6. Yazidi M, Abid A, Bel Hadj Sliman C, et al. Lipid profile in primary aldosteronism: cross-sectional and post-treatment analyses. Lipids. 2026 Mar 17. doi:10.1002/lipd.70051. Online ahead of print.

7. Xu M, Wei Y, Li M, et al. Primary aldosteronism results in a decline in estimated glomerular filtration rate independent of blood pressure: a case-control study. Kidney Blood Press Res. 2025;50:656-668. doi:10.1159/000547760.

8. Xu Z, Yang J, Hu J, et al. Primary aldosteronism in patients in China with recently detected hypertension. J Am Coll Cardiol. 2020;75:1913-1922. doi:10.1016/j.jacc.2020.02.052.

9. Xie W, Lin R, Meng Y, et al. DPA/AA ratio as a potential biomarker of primary aldosteronism in young and middle-aged adults. Front Endocrinol (Lausanne). 2026;17:1771955. doi:10.3389/fendo.2026.1771955.

10. Wu S, Li S, Huang J, et al. The association between blood pressure variability and renal damage in patients with primary aldosteronism. J Clin Hypertens (Greenwich). 2024;26:765-771. doi:10.1111/jch.14824.

11. Willenberg HS, Kolentini C, Quinkler M, et al. The serum sodium to urinary sodium to (serum potassium)2 to urinary potassium (SUSPPUP) ratio in patients with primary aldosteronism. Eur J Clin Invest. 2009;39:43-50. doi:10.1111/j.1365-2362.2008.02060.x.

12. Sun K, Gong M, Yu Y, et al. Diagnostic accuracy of 24-hour urinary aldosterone for primary aldosteronism in northeast China. J Clin Hypertens (Greenwich). 2025;27:e70130. doi:10.1111/jch.70130.

13. Tang J, Tan L, Zhang T, et al. Intraindividual variability of aldosterone and the aldosterone-to-renin ratio detected by LC-MS/MS and their impact factors in patients with hypertension. Endocrine. 2026;91:50. doi:10.1007/s12020-025-04481-8.

14. Watanabe D, Morimoto S, Morishima N, Ichihara A. Comparison of the clinical significance of lymphocyte-based inflammatory indices between the two major subtypes of primary aldosteronism. Endocrine. 2025;88:863-872. doi:10.1007/s12020-025-04193-z.

15. Rossi GP, Bernini G, Desideri G, et al. Renal damage in primary aldosteronism: results of the PAPY Study. Hypertension. 2006;48:232-238. doi:10.1161/01.HYP.0000230444.01215.6A.

16. Qin S, Li J, Wu S, et al. Risk factors for renal impairment in primary aldosteronism: a retrospective study. Front Endocrinol (Lausanne). 2025;16:1606182. doi:10.3389/fendo.2025.1606182.

17. Lv X, Hu H, Shen C, et al. Risk factors associated with lower bone mineral density in primary aldosteronism patients. Front Endocrinol (Lausanne). 2022;13:884302. doi:10.3389/fendo.2022.884302.

18. Laney M, Nabi-Bourgois A, Mallart-Riancho J, et al. Measurement accuracy and cutoffs for predicting primary aldosteronism diagnosis using Lumipulse for renin and aldosterone measurements. PLoS One. 2025;20:e0319219. doi:10.1371/journal.pone.0319219.

19. Kishimoto S, Matsumoto T, Oki K, et al. Microvascular endothelial function is impaired in patients with idiopathic hyperaldosteronism. Hypertens Res. 2018;41:932-938. doi:10.1038/s41440-018-0093-6.

20. Iwakura Y, Ito S, Morimoto R, et al. Renal resistive index predicts postoperative blood pressure outcome in primary aldosteronism. Hypertension. 2016;67:654-660. doi:10.1161/HYPERTENSIONAHA.115.05924.

21. Huang X, Yu S, Xiao H, et al. Comparison of clinical features between primary aldosteronism and essential hypertension in Chinese patients: a case-control study. Int J Endocrinol. 2021;2021:6685469. doi:10.1155/2021/6685469.

22. Wang D, Xu JZ, Chen X, et al. Speckle-tracking echocardiographic layer-specific strain analysis on subclinical left ventricular dysfunction in patients with primary aldosteronism. Am J Hypertens. 2019;32:155-162. doi:10.1093/ajh/hpy175.

23. Haze T, Hatakeyama M, Komiya S, et al. Association of the ratio of visceral-to-subcutaneous fat volume with renal function among patients with primary aldosteronism. Hypertens Res. 2021;44:1341-1351. doi:10.1038/s41440-021-00719-w.

24. Chen S, Chen K, Wu J, et al. Diagnostic value of 24-h urinary aldosterone and related biomarkers for screening primary aldosteronism in medication-exposed populations. Front Endocrinol (Lausanne). 2026;17:1792072. doi:10.3389/fendo.2026.1792072.

25. Iacobellis G, Petramala L, Marinelli C, et al. Epicardial fat thickness and primary aldosteronism. Horm Metab Res. 2016;48:238-241. doi:10.1055/s-0035-1559769.

26. Moon SJ, Jang HN, Kim JH, et al. Lipid profiles in primary aldosteronism compared with essential hypertension: propensity-score matching study. Endocrinol Metab (Seoul). 2021;36:885-894. doi:10.3803/EnM.2021.1012.

27. Concistrè A, Petramala L, Bonvicini M, et al. Comparisons of skin microvascular changes in patients with primary aldosteronism and essential hypertension. Hypertens Res. 2020;43:1222-1230. doi:10.1038/s41440-020-0475-4.

28. Choudhary MK, Värri E, Matikainen N, et al. Primary aldosteronism: higher volume load, cardiac output and arterial stiffness than in essential hypertension. J Intern Med. 2021;289:29-41. doi:10.1111/joim.13115.

29. Savard S, Amar L, Plouin PF, Steichen O. Cardiovascular complications associated with primary aldosteronism: a controlled cross-sectional study. Hypertension. 2013;62:331-336. doi:10.1161/HYPERTENSIONAHA.113.01060.

30. Kawashima A, Sone M, Inagaki N, et al. Renal impairment is closely associated with plasma aldosterone concentration in patients with primary aldosteronism. Eur J Endocrinol. 2019;181:339-350. doi:10.1530/EJE-19-0047.

31. Wu VC, Yang SY, Lin JW, et al. Kidney impairment in primary aldosteronism. Clin Chim Acta. 2011;412:1319-1325. doi:10.1016/j.cca.2011.02.018.

32. Kang B, Peng C, Wang K, et al. Elevated myoglobin in patients with primary aldosteronism: a cross-sectional study. Front Endocrinol (Lausanne). 2022;13:799174. doi:10.3389/fendo.2022.799174.

33. Fogari R, Preti P, Zoppi A, et al. Prevalence of primary aldosteronism among unselected hypertensive patients: a prospective study based on the use of an aldosterone/renin ratio above 25 as a screening test. Hypertens Res. 2007;30:111-117. doi:10.1291/hypres.30.111.

34. Šomlóová Z, Widimský J Jr, Rosa J, et al. The prevalence of metabolic syndrome and its components in two main types of primary aldosteronism. J Hum Hypertens. 2010;24:625-630. doi:10.1038/jhh.2010.65.

35. Turchi F, Ronconi V, Di Tizio V, et al. Primary aldosteronism and essential hypertension: assessment of cardiovascular risk at diagnosis and after treatment. Nutr Metab Cardiovasc Dis. 2014;24:476-482. doi:10.1016/j.numecd.2013.09.009.

36. Ohno Y, Sone M, Inagaki N, et al. Prevalence of cardiovascular disease and its risk factors in primary aldosteronism: a multicenter study in Japan. Hypertension. 2018;71:530-537. doi:10.1161/HYPERTENSIONAHA.117.10263.

37. Monticone S, Burrello J, Tizzani D, et al. Prevalence and clinical manifestations of primary aldosteronism encountered in primary care practice. J Am Coll Cardiol. 2017;69:1811-1820. doi:10.1016/j.jacc.2017.01.052.

38. Luo Q, Li NF, Yao XG, et al. Potential effects of age on screening for primary aldosteronism. J Hum Hypertens. 2016;30:53-61. doi:10.1038/jhh.2015.21.

39. Murata M, Kitamura T, Tamada D, et al. Plasma aldosterone level within the normal range is less associated with cardiovascular and cerebrovascular risk in primary aldosteronism. J Hypertens. 2017;35:1079-1085. doi:10.1097/HJH.0000000000001251.

40. Kobayashi H, Haketa A, Ueno T, et al. Plasma adrenocorticotropic hormone but not aldosterone is correlated with blood pressure in patients with aldosterone-producing adenomas. J Clin Hypertens (Greenwich). 2017;19:280-286. doi:10.1111/jch.12956.

41. Rosa J, Šomlóová Z, Petrák O, et al. Peripheral arterial stiffness in primary aldosteronism. Physiol Res. 2012;61:461-468. doi:10.33549/physiolres.932344.

42. Matsumura K, Fujii K, Oniki H, et al. Role of aldosterone in left ventricular hypertrophy in hypertension. Am J Hypertens. 2006;19:13-18. doi:10.1016/j.amjhyper.2005.05.013.

43. Mulatero P, Monticone S, Bertello C, et al. Long-term cardio- and cerebrovascular events in patients with primary aldosteronism. J Clin Endocrinol Metab. 2013;98:4826-4833. doi:10.1210/jc.2013-2805.

44. Muiesan ML, Salvetti M, Paini A, et al. Inappropriate left ventricular mass in patients with primary aldosteronism. Hypertension. 2008;52:529-534. doi:10.1161/HYPERTENSIONAHA.108.114140.

45. Jiang Y, Zhang C, Ye L, et al. Factors affecting parathyroid hormone levels in different types of primary aldosteronism. Clin Endocrinol (Oxf). 2016;85:267-274. doi:10.1111/cen.12981.

46. Galetta F, Bernini G, Franzoni F, et al. Cardiac remodeling in patients with primary aldosteronism. J Endocrinol Invest. 2009;32:739-745. doi:10.3275/6380.

47. Pimenta E, Gordon RD, Ahmed AH, et al. Cardiac dimensions are largely determined by dietary salt in patients with primary aldosteronism: results of a case-control study. J Clin Endocrinol Metab. 2011;96:2813-2820. doi:10.1210/jc.2011-0354.

48. Pilz S, Kienreich K, Gaksch M, et al. Aldosterone to active renin ratio as screening test for primary aldosteronism: reproducibility and influence of orthostasis and salt loading. Horm Metab Res. 2014;46:427-432. doi:10.1055/s-0034-1367033.

49. Zhang Z, Luo Q, Tuersun T, et al. Higher prevalence of metabolic disorders in patients with bilateral primary aldosteronism than unilateral primary aldosteronism. Clin Endocrinol (Oxf). 2021;94:3-11. doi:10.1111/cen.14318.

50. Yin GS, Zhang SL, Yan L, et al. New index of using serum sodium and potassium and urine sodium and potassium jointly in screening primary aldosteronism in hypertensive patients [Chinese]. Zhonghua Yi Xue Za Zhi. 2010;90:962-966.

51. Yamashita T, Shimizu S, Koyama M, et al. Screening of primary aldosteronism by clinical features and daily laboratory tests: combination of urine pH, sex, and serum K+. J Hypertens. 2018;36:326-334. doi:10.1097/HJH.0000000000001511.

52. Wu VC, Lo SC, Chen YL, et al. Endothelial progenitor cells in primary aldosteronism: a biomarker of severity for aldosterone vasculopathy and prognosis. J Clin Endocrinol Metab. 2011;96:3175-3183. doi:10.1210/jc.2011-1135.

53. van den Berg TNA, Thijssen DHJ, van Mil ACCM, et al. Plasma levels of the cardiovascular protective endogenous nucleoside adenosine are reduced in patients with primary aldosteronism without affecting ischaemia-reperfusion injury: a prospective case-control study. Eur J Clin Invest. 2019;49:e13180. doi:10.1111/eci.13180.

54. Tian S, Yu F, Zhang W, et al. Analysis of metabolic indexes and other characteristics in patients with primary aldosteronism [Chinese]. Clin Med China. 2022;38(1):62-67. doi:10.3760/cma.j.cn101721-20211015-000184.

55. Sechi LA, Novello M, Lapenna R, et al. Long-term renal outcomes in patients with primary aldosteronism. JAMA. 2006;295:2638-2645. doi:10.1001/jama.295.22.2638.

56. Sang M, Fu Y, Wei C, et al. Comparison of biomarkers of endothelial dysfunction and microvascular endothelial function in patients with primary aldosteronism and essential hypertension. J Renin Angiotensin Aldosterone Syst. 2021;22:1470320321999491. doi:10.1177/1470320321999491.

57. Ribstein J, Du Cailar G, Fesler P, Mimran A. Relative glomerular hyperfiltration in primary aldosteronism. J Am Soc Nephrol. 2005;16:1320-1325. doi:10.1681/ASN.2004100878.

58. Lottspeich C, Köhler A, Czihal M, et al. Atherosclerotic burden and arterial stiffness are not increased in patients with milder forms of primary aldosteronism compared to patients with essential hypertension. Horm Metab Res. 2021;53:178-184. doi:10.1055/a-1326-2164.

59. Liu HL, Zeng QT, Xu YY, et al. Association of plasma aldosterone concentration with early renal injury biomarkers in primary aldosteronism: a propensity-matched comparative study. J Clin Hypertens (Greenwich). 2025;27:e70124. doi:10.1111/jch.70124.

60. Irita J, Okura T, Manabe S, et al. Plasma osteopontin levels are higher in patients with primary aldosteronism than in patients with essential hypertension. Am J Hypertens. 2006;19:293-297. doi:10.1016/j.amjhyper.2005.08.019.

61. Hu W, Zhou PH, Zhang XB, et al. Pathophysiological functions of adrenomedullin and natriuretic peptides in patients with primary aldosteronism. Endocrine. 2015;48:661-668. doi:10.1007/s12020-014-0316-9.

62. Fallo F, Della Mea P, Sonino N, et al. Adiponectin and insulin sensitivity in primary aldosteronism. Am J Hypertens. 2007;20:855-861. doi:10.1016/j.amjhyper.2007.03.012.

63. Deng YJ, Zhang SL, Liu PM, et al. Comparison on metabolic disorders and uric acid levels between patients with primary aldosteronism and essential hypertension [Chinese]. Zhonghua Xin Xue Guan Bing Za Zhi. 2016;44(9):743-749. doi:10.3760/cma.j.issn.0253-3758.2016.09.003.

64. Steichen O, Blanchard A, Plouin PF. Assessment of serum sodium to urinary sodium divided by (serum potassium)2 to urinary potassium as a screening tool for primary aldosteronism. Eur J Clin Invest. 2011;41:189-194. doi:10.1111/j.1365-2362.2010.02401.x.

65. Pizzolo F, Chiecchi L, Morandini F, et al. Increased urinary excretion of the epithelial Na channel activator prostasin in patients with primary aldosteronism. J Hypertens. 2017;35:355-361. doi:10.1097/HJH.0000000000001168.

66. Liu G, Yin GS, Tang JY, et al. Endothelial dysfunction in patients with primary aldosteronism: a biomarker of target organ damage. J Hum Hypertens. 2014;28:711-715. doi:10.1038/jhh.2014.11.

67. Iacobellis G, Petramala L, Cotesta D, et al. Adipokines and cardiometabolic profile in primary hyperaldosteronism. J Clin Endocrinol Metab. 2010;95:2391-2398. doi:10.1210/jc.2009-2204.

68. Fernández-Argüeso M, Pascual-Corrales E, Bengoa Rojano N, et al. Higher risk of chronic kidney disease and progressive kidney function impairment in primary aldosteronism than in essential hypertension: case-control study. Endocrine. 2021;73:439-446. doi:10.1007/s12020-021-02704-2.

69. Catena C, Lapenna R, Baroselli S, et al. Insulin sensitivity in patients with primary aldosteronism: a follow-up study. J Clin Endocrinol Metab. 2006;91:3457-3463. doi:10.1210/jc.2006-0736.

70. Iwakura Y, Morimoto R, Kudo M, et al. Predictors of decreasing glomerular filtration rate and prevalence of chronic kidney disease after treatment of primary aldosteronism: renal outcome of 213 cases. J Clin Endocrinol Metab. 2014;99:1593-1598. doi:10.1210/jc.2013-2180.

71. Park KS, Kim JH, Yang YS, et al. Outcomes analysis of surgical and medical treatments for patients with primary aldosteronism. Endocr J. 2017;64:623-632. doi:10.1507/endocrj.EJ16-0530.

72. Wu VC, Kuo CC, Wang SM, et al. Primary aldosteronism: changes in cystatin C-based kidney filtration, proteinuria, and renal duplex indices with treatment. J Hypertens. 2011;29:1778-1786. doi:10.1097/HJH.0b013e3283495cbb.

73. Nakamura Y, Kobayashi H, Tanaka S, et al. Association between plasma aldosterone and markers of tubular and glomerular damage in primary aldosteronism. Clin Endocrinol (Oxf). 2021;94:920-926. doi:10.1111/cen.14434.

74. Yasuda H, Yokota K, Katabami T, et al. Different effect of aldosterone on corrected HCO3- across primary aldosteronism subtypes. Hypertens Res. 2026;49:1621-1631. doi:10.1038/s41440-026-02609-5.

**Table S2. NOS Quality Assessment for Case-Control Studies (n = 36)**

The NOS case-control form comprises four selection items (S1-S4), one comparability item (C; maximum 2 points), and three exposure items (E1-E3), for a maximum of 9 points. ★ = 1 point; ★★ = 2 points; — = 0 points. Scores ≥6 were treated as a descriptive threshold indicating higher methodological quality. Study numbers correspond to Table 1 of the main manuscript.

| **#** | **Study** | **S1 Case definition** | **S2 Representativeness of cases** | **S3 Selection of controls** | **S4 Definition of controls** | **C Comparability** | **E1 Ascertainment of exposure** | **E2 Same method of ascertainment** | **E3 Non-response rate** | **Total (/9)** |
| --- | --- | --- | --- | --- | --- | --- | --- | --- | --- | --- |
| 1 | Yang et al. 2017 | ★ | ★ | ★ | ★ | ★★ | ★ | ★ | — | **8** |
| 3 | Zhao et al. 2025 | ★ | ★ | ★ | ★ | ★★ | ★ | ★ | — | **8** |
| 4 | Zhang et al. 2020 | ★ | ★ | ★ | ★ | ★★ | ★ | ★ | — | **8** |
| 7 | Xu et al. 2025 | ★ | ★ | ★ | ★ | ★★ | ★ | ★ | — | **8** |
| 9 | Xie et al. 2026 | ★ | ★ | ★ | ★ | ★★ | ★ | ★ | — | **8** |
| 16 | Qin et al. 2025 | ★ | ★ | ★ | ★ | ★★ | ★ | ★ | — | **8** |
| 17 | Lv et al. 2022 | ★ | ★ | ★ | ★ | ★★ | ★ | ★ | — | **8** |
| 21 | Huang et al. 2021 | ★ | ★ | ★ | ★ | ★★ | ★ | ★ | — | **8** |
| 22 | Wang et al. 2019 | ★ | ★ | ★ | ★ | ★★ | ★ | ★ | — | **8** |
| 23 | Haze et al. 2021 | ★ | ★ | ★ | ★ | ★★ | ★ | ★ | — | **8** |
| 25 | Iacobellis et al. 2016 | ★ | ★ | ★ | ★ | ★ | ★ | ★ | — | **7** |
| 26 | Moon et al. 2021 | ★ | ★ | ★ | ★ | ★★ | ★ | ★ | — | **8** |
| 30 | Kawashima et al. 2019 | ★ | ★ | ★ | ★ | ★★ | ★ | ★ | — | **8** |
| 32 | Kang et al. 2022 | ★ | ★ | ★ | ★ | ★★ | ★ | ★ | — | **8** |
| 36 | Ohno et al. 2018 | ★ | ★ | ★ | ★ | ★★ | ★ | ★ | — | **8** |
| 40 | Kobayashi et al. 2017 | ★ | ★ | ★ | ★ | ★★ | ★ | ★ | — | **8** |
| 41 | Rosa et al. 2012 | ★ | ★ | ★ | ★ | ★★ | ★ | ★ | — | **8** |
| 42 | Matsumura et al. 2006 | ★ | ★ | ★ | ★ | ★ | ★ | ★ | — | **7** |
| 44 | Muiesan et al. 2008 | ★ | ★ | ★ | ★ | ★★ | ★ | ★ | — | **8** |
| 45 | Jiang et al. 2016 | ★ | ★ | ★ | ★ | ★★ | ★ | ★ | — | **8** |
| 46 | Galetta et al. 2009 | ★ | ★ | ★ | ★ | ★★ | ★ | ★ | — | **8** |
| 47 | Pimenta et al. 2011 | ★ | ★ | ★ | ★ | ★★ | ★ | ★ | — | **8** |
| 49 | Zhang et al. 2021 | ★ | ★ | ★ | ★ | ★★ | ★ | ★ | — | **8** |
| 53 | van den Berg et al. 2019 | ★ | ★ | ★ | ★ | — | ★ | ★ | — | **6** |
| 54 | Tian et al. 2022 | ★ | ★ | ★ | ★ | ★★ | ★ | ★ | — | **8** |
| 56 | Sang et al. 2021 | ★ | ★ | ★ | ★ | ★★ | ★ | ★ | — | **8** |
| 58 | Lottspeich et al. 2021 | ★ | ★ | ★ | ★ | ★★ | ★ | ★ | — | **8** |
| 59 | Liu et al. 2025 | ★ | ★ | ★ | ★ | ★★ | ★ | ★ | — | **8** |
| 60 | Irita et al. 2006 | ★ | ★ | ★ | ★ | ★ | ★ | ★ | — | **7** |
| 62 | Fallo et al. 2007 | ★ | ★ | ★ | ★ | ★★ | ★ | ★ | — | **8** |
| 63 | Deng et al. 2016 | ★ | ★ | ★ | ★ | ★★ | ★ | ★ | — | **8** |
| 65 | Pizzolo et al. 2017 | ★ | ★ | ★ | ★ | ★★ | ★ | ★ | — | **8** |
| 66 | Liu et al. 2014 | ★ | ★ | ★ | ★ | ★★ | ★ | ★ | — | **8** |
| 67 | Iacobellis et al. 2010 | ★ | ★ | ★ | ★ | ★★ | ★ | ★ | — | **8** |
| 68 | Fernández-Argüeso et al. 2021 | ★ | ★ | ★ | ★ | ★★ | ★ | ★ | — | **8** |
| 73 | Nakamura et al. 2021 | ★ | ★ | ★ | ★ | ★★ | ★ | ★ | — | **8** |

Note: Total scores were recalculated from the item-level ratings and cross-checked against the Appraisal result column of Table 1.

**Table S3. NOS Quality Assessment for Cohort Studies (n = 18)**

The NOS cohort form comprises four selection items (S1-S4), one comparability item (C; maximum 2 points), and three outcome items (O1-O3), for a maximum of 9 points. ★ = 1 point; ★★ = 2 points; — = 0 points. Scores ≥6 were treated as a descriptive threshold indicating higher methodological quality. Study numbers correspond to Table 1 of the main manuscript.

| **#** | **Study** | **S1 Representativeness of exposed cohort** | **S2 Selection of non-exposed cohort** | **S3 Ascertainment of exposure** | **S4 Outcome of interest not present at start** | **C Comparability** | **O1 Assessment of outcome** | **O2 Length of follow-up** | **O3 Adequacy of follow-up** | **Total (/9)** |
| --- | --- | --- | --- | --- | --- | --- | --- | --- | --- | --- |
| 2 | Zhou et al. 2021 | ★ | ★ | ★ | ★ | ★ | ★ | ★ | ★ | **8** |
| 8 | Xu et al. 2020 (CONPASS) | ★ | ★ | ★ | ★ | ★★ | ★ | ★ | ★ | **9** |
| 12 | Sun et al. 2025 | ★ | ★ | ★ | ★ | ★ | ★ | ★ | ★ | **8** |
| 13 | Tang et al. 2026 | ★ | ★ | ★ | ★ | ★★ | ★ | ★ | ★ | **9** |
| 20 | Iwakura et al. 2016 | ★ | ★ | ★ | ★ | ★★ | ★ | ★ | ★ | **9** |
| 24 | Chen et al. 2026 | ★ | ★ | ★ | ★ | ★ | ★ | — | ★ | **7** |
| 31 | Wu et al. 2011 | ★ | ★ | ★ | ★ | ★★ | ★ | ★ | ★ | **9** |
| 33 | Fogari et al. 2007 | ★ | ★ | ★ | ★ | ★ | ★ | ★ | ★ | **8** |
| 35 | Turchi et al. 2014 | ★ | ★ | ★ | ★ | ★ | ★ | ★ | ★ | **8** |
| 43 | Mulatero et al. 2013 | ★ | ★ | ★ | — | ★★ | ★ | ★ | ★ | **8** |
| 52 | Wu et al. 2011 EPC | ★ | ★ | ★ | ★ | ★ | ★ | — | ★ | **7** |
| 55 | Sechi et al. 2006 | ★ | ★ | ★ | ★ | ★ | ★ | ★ | ★ | **8** |
| 57 | Ribstein et al. 2005 | ★ | ★ | ★ | ★ | ★★ | ★ | ★ | ★ | **9** |
| 61 | Hu et al. 2015 | ★ | ★ | ★ | ★ | ★★ | ★ | ★ | ★ | **9** |
| 69 | Catena et al. 2006 | ★ | ★ | ★ | ★ | ★★ | ★ | ★ | ★ | **9** |
| 70 | Iwakura et al. 2014 | ★ | ★ | ★ | ★ | ★ | ★ | ★ | ★ | **8** |
| 71 | Park et al. 2017 | ★ | ★ | ★ | ★ | ★ | ★ | ★ | ★ | **8** |
| 72 | Wu CysC 2011 | ★ | ★ | ★ | ★ | ★★ | ★ | ★ | ★ | **9** |

Note: Total scores were recalculated from the item-level ratings and cross-checked against the Appraisal result column of Table 1.

**Table S4.** JBI Item-Level Assessment for Analytical Cross-Sectional Studies (n = 20)

The JBI analytical cross-sectional checklist comprises eight items: Q1, clearly defined sample inclusion criteria; Q2, detailed description of study participants and setting; Q3, valid and reliable exposure measurement; Q4, objective and standard criteria for measurement of the condition; Q5, identification of confounding factors; Q6, stated strategies to address confounding; Q7, valid and reliable outcome measurement; and Q8, appropriate statistical analysis. Each item was judged as Yes (Y), No (N), Unclear (U), or Not applicable (NA). No numerical total score or arbitrary high-quality cutoff was applied. Study numbers correspond to Table 1 of the main manuscript.

| **#** | **Study** | **Q1** | **Q2** | **Q3** | **Q4** | **Q5** | **Q6** | **Q7** | **Q8** |
| --- | --- | --- | --- | --- | --- | --- | --- | --- | --- |
| 5 | Yuan et al. 2023 | Y | Y | Y | Y | N | N | Y | Y |
| 6 | Yazidi et al. 2026 | Y | Y | Y | Y | Y | Y | Y | Y |
| 10 | Wu et al. 2024 | Y | Y | Y | Y | Y | Y | Y | Y |
| 11 | Willenberg et al. 2009 | Y | Y | Y | Y | Y | U | Y | Y |
| 14 | Watanabe et al. 2025 | Y | Y | Y | Y | Y | Y | Y | Y |
| 15 | Rossi et al. 2006 PAPY | Y | Y | Y | Y | Y | Y | Y | Y |
| 18 | Laney et al. 2025 | Y | Y | Y | Y | N | N | Y | Y |
| 19 | Kishimoto et al. 2018 | Y | Y | Y | Y | Y | Y | Y | Y |
| 27 | Concistrè et al. 2020 | Y | Y | Y | Y | Y | Y | Y | Y |
| 28 | Choudhary et al. 2021 | Y | Y | Y | Y | Y | Y | Y | Y |
| 29 | Savard et al. 2013 | Y | Y | Y | Y | Y | Y | Y | Y |
| 34 | Šomlóová et al. 2010 | Y | Y | Y | Y | Y | Y | Y | Y |
| 37 | Monticone et al. 2017 | Y | Y | Y | Y | Y | Y | Y | Y |
| 38 | Luo et al. 2016 | Y | Y | Y | Y | Y | Y | Y | Y |
| 39 | Murata et al. 2017 | Y | Y | Y | Y | Y | Y | Y | Y |
| 48 | Pilz et al. 2014 | Y | Y | Y | Y | Y | Y | Y | Y |
| 50 | Yin et al. 2010 | Y | Y | Y | Y | Y | Y | Y | Y |
| 51 | Yamashita et al. 2018 | Y | Y | Y | Y | Y | Y | Y | Y |
| 64 | Steichen et al. 2011 | Y | Y | Y | Y | Y | Y | Y | Y |
| 74 | Yasuda et al. 2026 | Y | Y | Y | Y | Y | Y | Y | Y |

Note: Y = Yes; N = No; U = Unclear; NA = Not applicable.

**Table S5.** Summary of Critical Appraisal for the 74 Included Studies

| **Appraisal category** | **Appraisal tool** | **k** | **Numerical summary** | **Appraisal result** |
| --- | --- | --- | --- | --- |
| NOS case-control form | NOS for case-control (maximum 9) | 36 | Range 6-8; median 8 | 36/36 met the stated threshold of ≥6 |
| NOS cohort form | NOS for cohort (maximum 9) | 18 | Range 7-9; median 8 | 18/18 met the stated threshold of ≥6 |
| Analytical cross-sectional studies | JBI 8-item analytical cross-sectional checklist | 20 | No numerical total or cutoff applied | Item-level Y/N/U/NA judgments are reported in Table S4 |

All 54 studies assessed using the NOS met the stated descriptive threshold of ≥6/9. The 20 analytical cross-sectional studies were reported using item-level JBI judgments, without an overall numerical score or universal cutoff. No study was excluded solely on the basis of critical appraisal.

**Table S6.** Approximate Back-Conversion of Pooled SMDs to Clinical Units

| **Outcome** | **Pooled SMD (95% CI)** | **Median pooled within-study SD** | **Approximate back-converted difference (95% CI)** | **Direct pooled MD sensitivity (95% CI)** |
| --- | --- | --- | --- | --- |
| Serum potassium | −1.15 (−1.27 to −1.02) | 0.428 mmol/L | −0.49 mmol/L (−0.54 to −0.44) | −0.49 mmol/L (−0.53 to −0.44) |
| Serum sodium | 0.51 (0.38 to 0.63) | 2.349 mmol/L | +1.19 mmol/L (+0.90 to +1.49) | +1.20 mmol/L (+0.90 to +1.50) |
| Serum uric acid | −0.23 (−0.35 to −0.12) | 95.005 μmol/L | −22.2 μmol/L (−33.0 to −11.4) | −20.7 μmol/L (−29.9 to −11.6) |

Note: For each outcome, a pooled within-group SD was calculated within each study from the two group-specific SDs and sample sizes. The median of these study-specific pooled SDs was then multiplied by the pooled Hedges' g and its 95% CI. These values are descriptive back-conversions and are not direct MD meta-analyses. Parallel random-effects MD sensitivity analyses were also performed after harmonizing units; their results are shown in the final column and in Figure S1.

**Table S7.** Study-level Hormone Measurements Reported by the Included Studies

Only studies with extractable two-group hormone values are shown. Values are presented as index group / comparator in the group order shown, with units retained as reported. Empty cells indicate unavailable extractable values. PRA and direct/active renin are presented separately.

| **Study** | **Comparison** | **PAC / aldosterone** | **PRA** | **Direct / active renin** | ARR |
| --- | --- | --- | --- | --- | --- |
| Ribstein et al. 2005 | PA vs EH | 39±5 SEM / 18±2 SEM ng/dL | 0.17±0.02 SEM / 1.23±0.31 SEM ng/mL/h |  |  |
| Irita et al. 2006 | PA vs EHT | 234.0 (170.3–404.5) / 112.5 (72.1–144.5) pg/mL | 0.17 (0.0–0.47) / 0.37 (0.12–1.19) ng/mL/h |  |  |
| Matsumura et al. 2006 | PA vs EH | 465.6±65.5 SEM / 67.7±5.8 SEM pg/mL | 0.24±0.05 SEM / 0.83±0.12 SEM ng/mL/h |  |  |
| Rossi et al. 2006 PAPY | APA/IHA vs primary hypertension (PH) | APA332±41; IHA303±31 / PH148±5 pmol/L | APA 0.65±0.19; IHA 0.50±0.13 / PH 1.62±0.13 ng/mL/h |  | APA 103±25; IHA 72±10 / PH 24±3 ratio |
| Sechi et al. 2006 | PA vs EH | 243±195 / 167±97 pg/mL |  | Active renin concentration: 4.8±6.6 / 9.2±11.3 pg/mL |  |
| Fallo et al. 2007 | PA vs low-renin EH | 39.7±14.2 / 24.1±10.6 ng/dL | 0.2±0.1 / 0.6±0.3 ng/mL/h |  | 254±149 / 48±34 ratio |
| Fogari et al. 2007 | PA vs EH | 13.6±6.2 / 8.1±3.8 ng/dL | 0.29±0.18 / 0.78±0.18 ng/mL/h |  | 48.6±18.4 / 17.3±6.9 ng/dL per ng/mL/h |
| Galetta et al. 2009 | PA vs EH | 41.7±4.9 / 25.2±3.9 ng/dL | 0.26±0.05 / 1.24±0.27 ng/mL/h |  | 333.7±61.7 / 32.6±6.5 ng/dL per ng/mL/h |
| Willenberg et al. 2009 | PA vs EH | 416.4±359.1 / 96.9±62.9 ng/L |  | 4.4±4.2 / 26.4±38.2 ng/L | 136.5±100.0 / 11.9±16.2 ratio |
| Steichen et al. 2010 | PA vs EH | 556 (407–779) / 236 (157–330) pmol/L |  | 3.0 (1.6–4.9) / 9.1 (4.3–17.2) mIU/L | 101 (73–143) / 23 (13–39) pmol/mIU |
| Yin et al. 2010 | PA vs EH | 2211±1414 / 705±459 pmol/L | 0.25±0.27 / 2.73±2.32 nmol/L/h |  | 12848 (4197–35344) / 261 (171–500) pmol/L per nmol/L/h |
| Šomlóová et al. 2010 | PA vs EH | 636 ± 396 / 220 ± 175 ng/L | 0.495 ± 0.369 / 2.86 ± 3.34 source-reported mg/L/h |  | 190 ± 178 / 13.4 ± 11.5 ratio |
| Pimenta et al. 2011 | PA vs EH |  |  |  | 52.5±45.8 / 9.7±8.5 ratio |
| Wu et al. 2011 | PA vs EH | 40.1±30.9 / 20.1±10.7 ng/dL | 1.07±3.04 / 5.43±8.57 ng/mL/h |  | log: 72.11±0.84 / 0.99±0.71 |
| Wu et al. 2011 EPC | PA vs EH; APA vs IHA | 47.4±36.8 / 24.2±16.4 ng/dL | 0.80±1.26 / 6.56±10.01 ng/mL/h |  | log: 2.1±0.8 / 1.0±0.8 |
| Rosa et al. 2012 | PA vs EH | 37.8 (26.6–51.3) / 13.2 (8.2–23.2) ng/dL | 0.51 (0.42–0.64) / 0.88 (0.64–1.27) |  | 74 (47–105) / 18 (9–24) |
| Mulatero et al. 2013 | PA vs EH | 29 (21–40.2) / 17.5 (11.3–25.9) ng/dL | 0.2 (0.1–0.3) / 1.7 (0.6–5.2) ng/mL/h |  | 135 (88–232) / 9.7 (3.6–26.8) ratio |
| Savard et al. 2013 | PA vs EH | 674.8±451.3 / 279.1±170.9 pmol/L |  | Active renin concentration: 3.8±3.4 / 27.5±138.1 mU/L | 122.3±84.5 / 29.5±21.1 ratio |
| Liu et al. 2014 | PA vs EH | 61.7±34.1 / 13.6±6.4 ng/dL | 0.3±0.3 / 1.7±1.5 ng/mL/h |  | 535.6 (28.9–1468) / 9.3 (0.4–128) ratio |
| Pilz et al. 2014 | PA vs EH | 30.2 (23.7–54.4) / 16.4 (12.3–23.4) ng/dL |  | Active renin concentration: 3.1 (2.1–4.9) / 10.9 (5.1–26.7) μU/mL | 8.37 (5.0–14.85) / 1.6 (0.63–3.08) ng/dL per μU/mL |
| Turchi et al. 2014 | PA vs EH | 51.7±34.1 / 16.6±11.1 ng/dL | 0.4 (0.2–0.7) / 1.6±1.4 ng/mL/h |  | 104.6 (54–180) / 19.9±18.5 ratio |
| Hu et al. 2015 | PA vs low-renin EH | 398±92 / 307±84 pmol/L | 0.49±0.21 / 0.52±0.31 ng/mL/h |  | 51±6 / 32±4 ng/dL per ng/mL/h |
| Iwakura et al. 2016 | APA vs EH | 28±1.6 SEM / 16.2±0.8 SEM ng/dL | 0.8±0.2 SEM / 3±0.5 SEM ng/mL/h |  | 149.6±16.6 SEM / 19.3±3.4 SEM ratio |
| Jiang et al. 2016 | PA vs primary hypertension | 1347 (945–1893) / 584 (448–798) pmol/L | 0.2 (0.1–0.4) / 0.6 (0.3–2.0) nmol/L/h |  |  |
| Luo et al. 2016 | PA vs EH/non-PA | 24.0±21.9 / 12.9±6.5 ng/dL | 0.42±0.23 / 1.49±1.14 ng/mL/h |  | 55.9 (36.2–80.5) / 11 (6.2–18.0) ratio |
| Monticone et al. 2017 | PA vs apparent EH | 31 (22–42.9) / 18.4 (12.5–26) ng/dL | 0.3 (0.2–0.5) / 1.6 (0.8–2.6) ng/mL/h |  | 102 (64–166) / 12 (7–21) ng/dL per ng/mL/h |
| Pizzolo et al. 2017 | PA vs low-renin EH | Geometric mean 334 (95% CI296–376) / 157 (142–173) pg/mL |  | Direct/active renin: Direct renin 3.90 (2.65–5.74) / 5.07 (3.94–6.52) | 88.02 (69.04–112.21) / 32.0 (24.85–41.20) |
| Yang et al. 2017 | PA vs EH | 346±243 / 180±107 pg/mL | 0.87±1.04 / 3.18±2.56 ng/mL/h |  | log10: 2.84±0.64 / 1.95±0.65 |
| Kishimoto et al. 2018 | Pooled PA vs EH | 25.9 ± 13.7 / 12.4 ± 4.4 ng/dL | 0.45 ± 0.355 / 2 ± 2.1 ng/mL/h |  | 88.9 ± 85.8 / 13 ± 16.7 ratio |
| Kawashima et al. 2019 | PA vs EH | 15.7 (11.8–25.2) / 14.9 (11.2–18.0) ng/dL | 0.3 (0.2–0.6) / 2.4 (1.4–4.6) ng/mL/h |  | 46 (30–91) / 6.5 (3.6–10.3) ratio |
| van den Berg et al. 2019 | PA vs EHT (active-renin subset for ARR) | 0.81 (0.61–0.93) / 0.22 (0.11–0.28) nmol/L |  |  | 0.22 (0.17–0.25) / 0.01 (0.01–0.02) nmol/mU |
| Concistrè et al. 2020 | PA vs EH | 324.1±156 / 105±55.5 |  |  | 77.3±60.2 / 8.3±8.4 ratio |
| Xu et al. 2020 (CONPASS) | PA vs EH |  |  | Direct renin concentration (PRC): 3.6 (1.1-6.6) / 14.0 (6.7-26.7) mIU/L | 45 (27-138) / 6.3 (3-12) ng/mIU |
| Zhang et al. 2020 | PA vs EH |  | 0.10 (0.03-0.26) / 1.76 (1.00-2.97) ng/mL/h |  | 343.75 (98.05-2224.17) / 7.57 (3.53-18.55) ratio |
| Fernández-Argüeso et al. 2021 | PA vs EH | 48.9±5.6 / 13.4±1.5 | 0.6±0.1 / 2.2±0.7 |  |  |
| Lottspeich et al. 2021 | PA vs EH | 128 (97–154) / 91 (66–110) ng/L |  | 2.1 (2.0–3.8) / 7.3 (2.0–21.2) mU/L |  |
| Moon et al. 2021 | PA vs EH | 28.2 (20.8–41.3) / 14.4 (7.5–18.8) ng/dL | 0.1 (0.1–0.3) / 2.3 (1.3–5.2) ng/mL/h |  | 198.8 (99.2–294.3) / 5.3 (2.7–10.2) ratio |
| Nakamura et al. 2021 | PA vs EH | 555 (419–741) / 330 (241–388) pmol/L | 0.5 (0.3–0.8) / 0.9 (0.7–2.2) µg/L/h |  |  |
| Zhou et al. 2021 | PA vs EH | 32±12 / 23±9 ng/dL | 0.2 (IQR 0.5) / 3.0 (IQR 2.7) ng/mL/h |  |  |
| Kang et al. 2022 | PA vs EH | 270 (192–401) / 116 (72.3–171) pg/mL |  | Direct renin concentration (PRC): 2.5 (0.9–7.0) / 12.8 (5.1–32.2) mIU/mL | 100.3 (33.2–319.2) / 7.2 (3.2–21.7) pg/mL per mIU/mL |
| Lv et al. 2022 | PA vs EH | 31.0 (26.8–49.8) / 21.8 (14.3–28.7) ng/dL | 0.18 (0.07–0.63) / 4.91 (1.59–8.33) ng/mL/h |  | 171.4 (64.9–1037.4) / 4.4 (2.7–12.1) ratio |
| Tian et al. 2022 | PA vs EH | 156.62 (66.53–730.29) / 155.48 (85.68–572.74) ng/L |  | 1.61 (0.34–7.23) / 12.81 (1.9–82.45) ng/L | 96.85 (26.06–506.1) / 13.22 (1.06–54.63) ratio |
| Yuan et al. 2023 | PA vs EH |  | 0.1 (0.1-0.2) / 0.7 (0.3-1.7) ng/mL/h |  | 94.9 (53.0-227.5) / 10.1 (2.9-19.8) ratio |
| Laney et al. 2025 | PA vs EH | 661 [463–859] / 272 [229–315] pmol/L |  | Direct renin concentration (PRC): 3.0 [1.9–4.0] / 14.6 [10.1–19.1] mIU/L | 127.9 [88.0–167.6] / 29.4 [23.0–36.1] pmol/mIU |
| Liu et al. 2025 | PA vs EH | 25.11 (19.38–33.25) / 17.66 (12.62–24.49) ng/dL | 0.17 (0.02–0.48) / 2.05 (0.93–4.17) ng/mL/h |  |  |
| Qin et al. 2025 | PA vs EH | 245.76±192.22 / 199.20±47.70 pg/mL |  |  |  |
| Watanabe et al. 2025 | APA/IHA/EH |  | APA 0.2 (0.1-0.4); IHA 0.4 (0.2-0.7) / EH 1.2 (0.8-2.1) ng/mL/h |  |  |
| Xu et al. 2025 | PA vs EH |  |  | Direct renin concentration (PRC): 2.72 (1.08-6.60) / 14.30 (7.59-30.73) pg/mL | 73.14 (36.66-203.05) / 9.61 (5.25-17.69) ratio |
| Chen et al. 2026 | PA vs EH | 17.7 (13.02–24.97) / 12.5 (8.25–17.92) ng/dL |  | 3.5 (1.15–6.65) / 17.75 (6.1–38.67) mIU/L | 5.25 (2.58–11.45) / 0.8 (0.2–1.8) ratio |
| Tang et al. 2026 | PA vs EH |  | 0.42 (0.19-0.65) / 1.06 (0.66-2.38) ng/mL/h |  | 40.44 (16.67-109.33) / 7.33 (4.26-11.94) ratio |
| Yasuda et al. 2026 | PA vs non-PA |  | 0.28 (0.20–0.46) / 0.8 (0.40–1.90) ng/mL/h |  | 399 (233–783) / 54.8 (20.6–112) ratio |
| Yazidi et al. 2026 | PA vs EH |  |  |  | 87.6 (48.7-165.5) / 12.9 (7.5-17.7) study-specific |

**Supplementary Figures**

**Figure S1 Clinical-unit mean-difference sensitivity analyses.**

Panels show random-effects mean-difference analyses after harmonizing the original measurement units for (A) serum potassium, (B) serum sodium, and (C) serum uric acid. The pooled PA-minus-hypertensive-control differences were −0.49 mmol/L (95% CI: −0.53 to −0.44), +1.20 mmol/L (95% CI: +0.90 to +1.50), and −20.7 μmol/L (95% CI: −29.9 to −11.6), respectively. These are directly pooled between-group MDs and are distinct from the descriptive SMD back-conversions in Table S6.

**Figure S2** Complete-data sensitivity analyses for serum potassium including Galetta et al. (2009).

Panels show (A) publication-year meta-regression using all 59 studies, (B) the corresponding funnel plot with Galetta et al. (2009) identified, and (C) trim-and-fill analysis. In the complete dataset, the potassium difference narrowed with publication year (slope = 0.042 per year, 95% CI: 0.024 to 0.061; p < 0.001; R² = 16.5%). Funnel-plot asymmetry was detected, whereas trim-and-fill imputed no studies and did not materially change the pooled estimate. These analyses complement the main-text sensitivity analyses excluding Galetta et al. (2009).

**Figure S3** Recruitment-source and sex-composition analyses.

Panels show (A) the recruitment-source subgroup analysis for serum potassium; complete-data meta-regression of the PA-minus-control difference in male proportion, per 10-percentage-point increment, for (B) serum potassium; the corresponding potassium sensitivity analysis excluding Galetta et al. 2009 in (C); and complete-data sex-composition meta-regression for (D) serum sodium, (E) serum creatinine, and (F) eGFR. The potassium association remained significant after exclusion of Galetta et al. 2009 (complete data: β = −0.274, 95% CI: −0.437 to −0.111, p < 0.001; excluding Galetta: β = −0.241, 95% CI: −0.396 to −0.086, p = 0.002). The sodium, creatinine, and eGFR slopes were not statistically significant. A corresponding recruitment-source subgroup analysis for serum sodium was not estimable because too few studies recruited participants from community or primary-care settings. All panels present study-level analyses and do not demonstrate individual-level sex effects.

**Figure S4** Publication-year meta-regression for outcomes other than serum potassium.

Bubble plots show publication-year meta-regression for (A) serum sodium, (B) serum creatinine, (C) eGFR, and (D) serum uric acid. Bubble area is proportional to inverse-variance weight, and the fitted line represents the mixed-effects meta-regression. No statistically significant temporal trend was identified for these outcomes; publication year was a significant moderator only for serum potassium in the main analysis.

**Figure S5** Geographic-region subgroup analyses for outcomes other than serum potassium.

Subgroup forest plots compare East-Asian and non-East-Asian studies for (A) serum sodium, (B) serum creatinine, (C) eGFR, and (D) serum uric acid. A regional subgroup analysis was not performed for BUN because all eligible BUN studies were conducted in East Asia. These study-level analyses explore geographic variation and should be interpreted in conjunction with the number of studies and residual heterogeneity within each subgroup.

**Figure S6** Additional urinary-protein and acid-base outcomes.

Panels show (A) 24-hour urinary albumin excretion analyzed as a mean difference, (B) total urinary protein or UPCR analyzed separately from albumin-specific outcomes, (C) directly measured bicarbonate, (D) standard calculated bicarbonate, and (E) potassium-adjusted calculated bicarbonate. Directly measured bicarbonate was reported by one study (MD = 3.00 mmol/L); two studies reporting standard calculated bicarbonate yielded a pooled MD of 0.79 mmol/L (95% CI: -0.34 to 1.93); and one study reported potassium-adjusted calculated bicarbonate (MD = 0.85 mmol/L). These acid-base measures were not pooled together.

**Figure S7** Additional strict PA-subtype comparisons.

Forest plots compare unilateral/lateralizing PA with bilateral PA for (A) serum creatinine and (B) eGFR, using the strict subtype dataset based preferentially on adrenal venous sampling, surgery, or pathology. Serum creatinine did not differ significantly (13 studies; pooled SMD = -0.07, 95% CI: -0.24 to 0.10), and eGFR was also similar between subtypes (6 studies; pooled SMD = 0.05, 95% CI: -0.09 to 0.19).

**Figure S8** Expanded PA-subtype sensitivity analyses.

Forest plots show expanded subtype analyses for (A) serum potassium, (B) serum sodium, (C) serum creatinine, and (D) eGFR after adding studies classified by imaging-only or other sensitivity-only subtype methods to the strict subtype dataset. These analyses evaluate whether the primary subtype findings depend on the method used to assign unilateral/lateralizing and bilateral PA.

**Figure S9** Overall hormone-profile forest plots: PA versus hypertensive controls.

Separate forest plots summarize compatible study-level comparisons for PAC, PRA, direct renin concentration, raw ARR, log-transformed ARR, and urinary aldosterone. PA was characterized by higher PAC and ARR and lower renin measures. PRA and direct renin were not combined, raw and log-transformed ARR were analyzed separately, and urinary aldosterone was retained as a distinct outcome because assays and reporting scales differed across studies.

**Figure S10** Hormone-profile forest plots for PA-subtype comparisons.

Panels show (A) PAC in the strict subtype dataset, (B) PAC in the expanded subtype sensitivity dataset, (C) PRA in the strict subtype dataset, (D) PRA in the expanded subtype sensitivity dataset, (E) ARR in the expanded subtype sensitivity dataset, and (F) urinary aldosterone in the strict subtype dataset. Unilateral/lateralizing PA was generally characterized by higher PAC and ARR and lower PRA than bilateral PA. The urinary-aldosterone comparison was based on one study and was not quantitatively pooled.

**Figure S11** Study-level hormone-outcome meta-regression analyses.

Panels show associations of mean PA-group PAC with the effect sizes for (A) serum potassium, (B) serum sodium, and (C) eGFR; PRA-suppression analyses for potassium using (D) complete data and (E) data excluding Galetta et al. 2009, and for (F) serum sodium; and between-group raw ARR-contrast analyses for potassium using (G) complete data and (H) data excluding Galetta et al. 2009, for (I) serum sodium, and for (J) eGFR. Continuous PAC was not significantly associated with these outcomes. In complete-data potassium models, PRA suppression and ARR contrast were associated with more negative potassium SMDs (β = −0.737, p < 0.001; β = −0.245, p = 0.009), but neither association remained after Galetta et al. 2009 was excluded (β = 0.171, p = 0.473; β = −0.018, p = 0.842). For sodium, negative slopes were observed for PRA suppression (β = −0.494, p < 0.001) and ARR contrast (β = −0.207, p = 0.019), indicating smaller positive sodium SMDs as the moderator increased. ARR contrast was not associated with eGFR (p = 0.250). A positive PRA-suppression contrast denotes greater renin suppression in PA, and a positive ARR contrast denotes a larger PA-minus-control ARR separation. These study-level findings cannot establish patient-level causality.

**Figure S12** Leave-one-out sensitivity analyses for the principal outcomes.

Panels show leave-one-out analyses for (A) serum potassium (k = 59), (B) serum sodium (k = 31), (C) serum creatinine (k = 42), (D) eGFR (k = 36), (E) BUN (k = 8), and (F) serum uric acid (k = 23). Each row presents the pooled effect after omission of the indicated study. The direction and statistical interpretation of the potassium, sodium, creatinine, eGFR, and uric-acid results were not materially altered by removal of any single study. The BUN analysis should be interpreted as exploratory because of the small number of studies.

**Figure S13** Sensitivity analyses excluding studies with low-renin essential-hypertension comparators.

Forest plots show PA versus hypertensive-control comparisons after exclusion of low-renin essential-hypertension studies for (A) serum potassium, (B) serum sodium, (C) serum creatinine, and (D) BUN. The corresponding pooled SMDs were approximately -1.11, 0.49, 0.00, and -0.08, respectively, and remained consistent with the primary analyses. These analyses assess whether low-renin selection materially influenced the overall comparisons.

**Figure S14** Comparator-type subgroup analyses.

Subgroup forest plots compare ordinary or PA-screening-negative essential-hypertension comparators with low-renin essential-hypertension comparators for (A) serum potassium, (B) serum sodium, and (C) serum creatinine. Between-subgroup differences were significant for serum potassium (P = 0.002), but not for serum sodium (P = 0.264) or serum creatinine (P = 0.445). These study-level findings should be interpreted cautiously because the low-renin comparator subgroup contained few studies.

**Figure S15** Supplementary PAC-stratified analyses.

Panels show (A) the serum-potassium PAC subgroup sensitivity analysis after additionally including studies with median or converted PAC values, and reviewer-requested exploratory PAC subgroup analyses for (B) serum sodium, (C) eGFR, and (D) UACR/ACR. High and low PAC were defined using a study-level mean PAC threshold of 30 ng/dL. These categorical analyses describe study-level severity patterns and do not establish an individual-level dose-response relationship.
